# Elucidating the MultiPathway Therapeutic Potential of *Trichosanthes* *kirilowii* Bioactives in Intestinal Obstruction via Network Pharmacology and Molecular Dynamics

**DOI:** 10.1002/open.70312

**Published:** 2026-10-05

**Authors:** Tianying Wu, Xiaofen Lin, Wencong Lin, Lixia Huang, Yuewu Lin

**Affiliations:** ^1^ Department of Traditional Chinese Medicine Zhangzhou Affiliated Hospital of Fujian Medical University Zhangzhou China; ^2^ Department of Geriatrics Zhangzhou Affiliated Hospital of Fujian Medical University Zhangzhou China; ^3^ Department of Oncology Zhangzhou Affiliated Hospital of Fujian Medical University Zhangzhou China; ^4^ Department of Nuclear Medicine Zhangzhou Affiliated Hospital of Fujian Medical University Zhangzhou China

**Keywords:** intestinal obstruction, molecular docking, molecular dynamics, network pharmacology, *Trichosanthes kirilowii*

## Abstract

Intestinal obstruction (IO) disrupts normal intestinal transit and involves dysmotility, mucosal stress, and inflammation. This computational workflow evaluated a 10‐compound *Trichosanthes kirilowii* docking panel, a filtered disease‐target set, MOE docking validation, and 100 ns MOE molecular dynamics and MM/GBSA endpoint analyses. GeneCards filtering (relevance >= 10) and OMIM curation yielded a primary disease union of 756 genes; 31 targets overlapped with a broadened 203‐target herb‐associated set, and the overlap was significant by hypergeometric testing (*p* = 6.38 × 10^−11^). Docking across ACTB, AKT1, ALB, IL6, MYC, PTGS2, and TNFR1/TNFRSF1A showed strongest overall performance for β‐D‐glucopyranosyl‐α‐spinasterol (mean score −8.19 kcal/mol), followed by Stigmast‐7‐en‐3β‐ol 3‐O‐β‐D‐glucopyranoside, Cucurbitacin B, Bryonolic acid, and Cucurbitacin D. Redocking validation passed for ACTB, AKT1, ALB, PTGS2, and TNFR1, while IL6 and MYC were evaluated using alternative site validation. These results support a multicompound, multitarget hypothesis requiring experimental confirmation.

## Graphical abstract

Network pharmacology identifies 31 shared targets linking *T. kirilowii* bioactives to intestinal obstruction, highlighting TNF, PI3K‐Akt, and MAPK signaling. Comparative docking, validation, and 100 ns molecular dynamics with MM/GBSA nominate β‐D‐glucopyranosyl‐α‐spinasterol as the leading candidate, binding albumin with the strongest predicted affinity (−55.9 ± 5.6 kcal/mol). These convergent computational findings propose a multitarget therapeutic hypothesis warranting experimental validation.

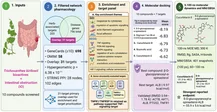

## 1 Introduction

Intestinal obstruction (IO) is a serious condition that disrupts the normal passage of food, fluids, and gas through the intestines, often requiring conventional treatments such as surgery or pharmacological interventions. However, these treatments may not provide long‐term relief, especially in chronic cases, prompting interest in alternative therapies like Traditional Chinese Medicine (TCM), which aims to improve gastrointestinal motility, reduce inflammation, and resolve obstructions [1, 2]. *Trichosanthes kirilowii*, a commonly used herb in TCM, is believed to promote bowel movements and alleviate constipation, making it a potential remedy for IO. Despite its widespread clinical use, the molecular mechanisms by which *T. kirilowii* exerts its effects are not well understood [3].

Intestinal obstruction is a clinically important cause of acute abdominal presentation and hospitalization. Small bowel obstruction is commonly associated with previous abdominal or pelvic surgery, postoperative adhesions, hernia, malignancy, inflammatory bowel disease, volvulus, and stricturing lesions; adhesive small bowel obstruction is especially common after abdominal operations. In high‐risk postoperative cohorts, approximately one in ten patients develops at least one episode of small bowel obstruction within three years after colectomy [4]. These risk factors create a mechanistic background in which inflammation, altered motility, epithelial stress, and tissue remodeling can contribute to obstruction‐related pathology [4, 5].

Previous experimental studies suggest that *T. kirilowii* and related constituents may influence gastrointestinal function through modulation of intestinal transit in constipation‐related models, attenuation of inflammatory mediators, improvement of epithelial‐barrier‐associated responses, and regulation of gut microbial composition [3, 6, 7, 8, 9]. These findings provide a biological rationale for exploring whether chemically defined *T. kirilowii* constituents interact with obstruction‐associated inflammatory and motility‐related targets.

This study therefore combined network pharmacology with a comparative molecular‐docking screen of ten named *T. kirilowii* constituents. The screen was designed to evaluate chemical diversity rather than prespecify a two‐compound mechanism. Cucurbitacin E and β‐D‐glucopyranosyl‐α‐spinasterol were subsequently advanced as lead compounds to 100 ns molecular dynamics and MM/GBSA analyses on the basis of the comparative docking results. Seven proteins were selected to represent complementary intestinal‐obstruction mechanisms: TNFR1/TNFRSF1A for TNF signaling [10], IL6 for inflammatory cytokine signaling [11], ACTB for cytoskeletal and contractile integrity [12], ALB for transport and systemic homeostasis, PTGS2 for COX‐2/prostaglandin amplification in bowel obstruction [13], AKT1 for stress‐response and repair signaling, and MYC for growth and remodeling control [14]. This staged design separates broad compound prioritization from detailed dynamic evaluation and yields experimentally testable, target‐specific hypotheses.

To translate network‐level predictions into molecular evidence, we integrate computational binding analysis to test whether the major compounds can plausibly engage these targets at the structural level. Molecular docking is used to estimate binding modes and prioritize compound–protein pairs, while molecular dynamics simulations provide a time‐resolved view of complex stability and binding‐site behavior under defined simulation conditions. By linking network pharmacology with structure‐based modeling, this study aims to generate a coherent mechanistic picture of how *T. kirilowii* may act on multiple IO‐relevant pathways simultaneously. Ultimately, these findings are intended to support the integration of TCM‐derived bioactives into modern therapeutic strategies for gastrointestinal motility disorders by providing target‐focused, mechanism‐driven hypotheses that can guide future experimental validation.

Network pharmacology provides a rational framework for TCM because it maps relationships among herbal constituents, biological targets, and disease pathways [15]. Accordingly, the primary structural screen comprised ten named and chemically diverse *T. kirilowii* constituents, including cucurbitacins, phytosterols, a sterol glycoside, pentacyclic triterpenoids, and a flavonoid supported by the phytochemical literature [16, 17, 18]. The two lead compounds were selected only after comparative docking. The target panel spans inflammatory signaling (TNFR1/TNFRSF1A and IL6), cytoskeletal and contractile integrity (ACTB), systemic transport and homeostasis (ALB), prostaglandin‐associated inflammatory amplification (PTGS2), stress‐response and repair signaling (AKT1), and transcriptional control of growth and remode‐ling (MYC).

## 2 Methodology

### 2.1 Prediction of Target Genes for Bioactive Compounds in *T. kirilowii*

The target genes of the bioactive compounds in *T. kirilowii* were predicted using the TCMSP database [19]. Duplicate entries were removed, protein identifiers were mapped to Homo sapiens, and gene symbols were standardized using UniProt [20], yielding a nonredundant herb‐associated target set.

TCMSP and the curated ten‐compound panel were used to define a broadened, nonredundant herb‐associated target set of 203 genes.

### 2.2 Prediction of Target Genes in Intestinal Obstruction

For intestinal obstruction, disease‐associated genes were retrieved from GeneCards and OMIM [21, 22]. GeneCards raw disease hits [14] were filtered using a relevance‐score cutoff of  >= 10, yielding 698 primary targets. These were combined with 58 additional unique OMIM genes to produce a 756‐gene primary disease union.

The primary overlap contained 31 herb‐disease targets (hypergeometric *p*  =  6.38 × 10^−11^). A broader sensitivity disease union of 2980 targets yielded 65 overlapping targets (*p*  =  1.55 × 10^−9^), supporting robustness of the overlap after target‐set filtering.

### 2.3 Construction of Venn Diagram for Target Gene Overlap

To analyze the overlap between the target genes of *T. kirilowii* and those involved in intestinal obstruction, a Venn diagram was constructed using the Venny interactive tool [23]. This analysis identified the common genes retained for statistical overlap testing, PPI construction, enrichment analysis, and subsequent target prioritization.

### 2.4 Protein–Protein Interaction (PPI) Network Analysis

The filtered 31‐target overlap network was first constructed using STRING, generating the complete PPI network with 28 mapped nodes and 102 edges. This complete PPI network was subsequently imported into Cytoscape for hub gene prioritization and visualization of the topological outputs.

The 31 primary overlapping targets were imported into STRING using H. sapiens and a combined score threshold  >= 0.700. The resulting PPI network contained 28 mapped nodes and 102 edges and was analyzed in Cytoscape 3.10.3 using CytoHubba Degree, Maximum Clique Centrality (MCC), and Maximum Neighborhood Component (MNC) algorithms [24, ‍25]. The topological importance of nodes was evaluated using these three CytoHubba algorithms, and the top 10 genes ranked by each method were compared using Venn analysis to identify consensus hub genes. The docking panel was selected using a transparent mixed rule: AKT1, ALB, ACTB, IL6, MYC, and PTGS2 were retained because they appeared in the available CytoHubba top‐node outputs and were mechanistically linked to inflammation, motility, barrier stress, or remodeling; TNFR1/TNFRSF1A was retained as a TNF‐signaling receptor and disease‐mechanism target but is interpreted as pathway‐supported rather than as a topological top‐10 hub. The hub selection logic is summarized in Table S18A.

### 2.5 Gene Ontology and Pathway Enrichment Analysis

GO and KEGG enrichment analyses were performed using the filtered 31‐target primary overlap [26, 28]. Enriched terms were interpreted as mechanistically relevant or as generic/multifunctional annotations rather than treated as direct evidence of disease causation.

### 2.6 Molecular Docking and Molecular Dynamics Simulations

Molecular docking was performed using the Molecular Operating Environment (MOE, version 2022) within an established structure‐based screening framework [29]. The expanded panel included ten named *T. kirilowii* constituents: Cucurbitacin E, β‐D‐glucopyranosyl‐α‐spinasterol, Cucurbitacin B, Cucurbitacin D, α‐Spinasterol, Stigmast‐7‐en‐3β‐ol, Stigmast‐7‐en‐3β‐ol 3‐O‐β‐D‐glucopyranoside, Bryonolic acid, Karounidiol, and Chrysoeriol [16, 17, 18]. Target structures were ACTB (6NBW), AKT1 (4EJN), ALB (1E7F), IL6 (4NI9), MYC (6E16), PTGS2 (5F19), and TNFR1/TNFRSF1A (9S3J). Proteins were prepared with MOE QuickPrep and the Amber99 force field. This chemically diverse panel enabled comparative prioritization rather than selective testing of a single compound class.

The ligand structures were imported into MOE, protonated, assigned appropriate partial charges, and energy‐minimized before docking. Binding sites were defined within MOE on the basis of the experimentally resolved ligand‐binding region, when available, or by the Site Finder module together with the residues forming the predicted functional pocket. Site Finder identifies cavities using alpha‐sphere positions; the alpha spheres corresponding to the selected pocket were converted to dummy atoms and used to delimit the docking region. Thus, docking was performed within an MOE‐defined binding site rather than an external fixed rectangular grid‐box protocol. The site‐definition logic, control ligand information, and final pose/contact residues are now summarized target‐by‐target in Tables S16 and S16A.

Docking validation was performed by native‐ligand redocking when a directly comparable crystallographic control was available and by a site‐specific pose benchmark otherwise. Validation passed for ACTB/Latrunculin B in the resolved pocket (RMSD 1.78 Å), AKT1/0R4 (0.94 Å), ALB/lauric acid (DAO) in the matched fatty‐acid pocket (1.49 Å), the PTGS2/5F19 COX‐2 active‐site benchmark (1.27 Å), and TNFR1/TNFRSF1A/9S3J with A1JLJ (1.12 Å). IL6 and MYC were treated as alternative‐site validation cases because their structures did not provide directly comparable small‐molecule redocking controls.

The final Cucurbitacin E and β‐D‐glucopyranosyl‐α‐spinasterol complexes were subjected to 100 ns MOE molecular dynamics simulations using the Amber99 force field at 300 K. The 300 K setting was used as a standardized comparative simulation condition rather than as a direct model of human body temperature; because 310 K more closely approximates physiological temperature, the MD conclusions are interpreted comparatively and should be validated under physiological temperature or experimental conditions in future work. For trajectory visualization, the complete plotted RMSD, Rg, and SASA series were expressed on a physical 0–100 ns time axis. The Origin RMSD projects contained target‐specific configuration coordinates and frame counts; therefore, for each panel, the first and last displayed configurations were mapped linearly to 0 and 100 ns, respectively, while all original RMSD values were retained without smoothing or interpolation. MM/GBSA endpoint binding‐energy estimates were calculated within the MOE 2022/Amber99 framework from 401 snapshots extracted every 100 ps from the equilibrated 60–100 ns segment. The reported expression comprised van der Waals, electrostatic, generalized Born polar‐solvation, and nonpolar‐solvation terms; configurational entropy was not included. Consequently, these values are interpreted as comparative endpoint estimates rather than experimental binding affinities. Hydrogen‐ bond occupancy, pocket RMSF, and per‐residue energy decomposition were evaluated over the same endpoint segment where reliable exports were available. For TNFR1/TNFRSF1A, interpretation is restricted to the verified 9S3J/A1JLJ redocking result, final docking scores, hydrogen‐bond persistence, and complex‐level/component MM/GBSA estimates. Residue‐contact identities and per‐residue energy contributions are not reported because a verified final 9S3J export was unavailable.

Trajectories were analyzed for ligand RMSD, protein‐backbone RMSD, binding‐pocket RMSD, residue‐level RMSF, radius of gyration (Rg), solvent‐accessible surface area (SASA), and protein–ligand hydrogen‐bond persistence. Full 0–100 ns RMSD, Rg, and SASA traces were used to assess time‐dependent structural behavior. MM/GBSA endpoint binding‐energy estimates, component energies, hydrogen‐ bond occupancy, and per‐residue energy contributions were evaluated for the equilibrated 60–100 ns segment using MOE 2022/Amber99‐compatible analysis functions. The MM/GBSA estimates exclude configurational entropy and were interpreted comparatively, not as direct experimental binding affinities. Per‐residue values are reported only where reliable exports were available; missing values were not inferred.

## 3 Results

### 3.1 Filtered Target‐Overlap and PPI Network

After GeneCards relevance filtering and OMIM curation, the primary intestinal‐obstruction disease union contained 756 genes. The broadened herb‐associated set contained 203 targets, of which 31 overlapped with the filtered disease set. The overlap was significant against a 19,500‐gene background (hypergeometric *p*  =  6.38 × 10^−11^). STRING retained 28 mapped nodes and 102 edges from the primary overlap (Figure 1; Tables 1 and S17). TNFRSF1A was included among the 31 primary overlap genes and was retained as a pathway‐supported target rather than a CytoHubba top‐10 consensus hub.

**FIGURE 1:**
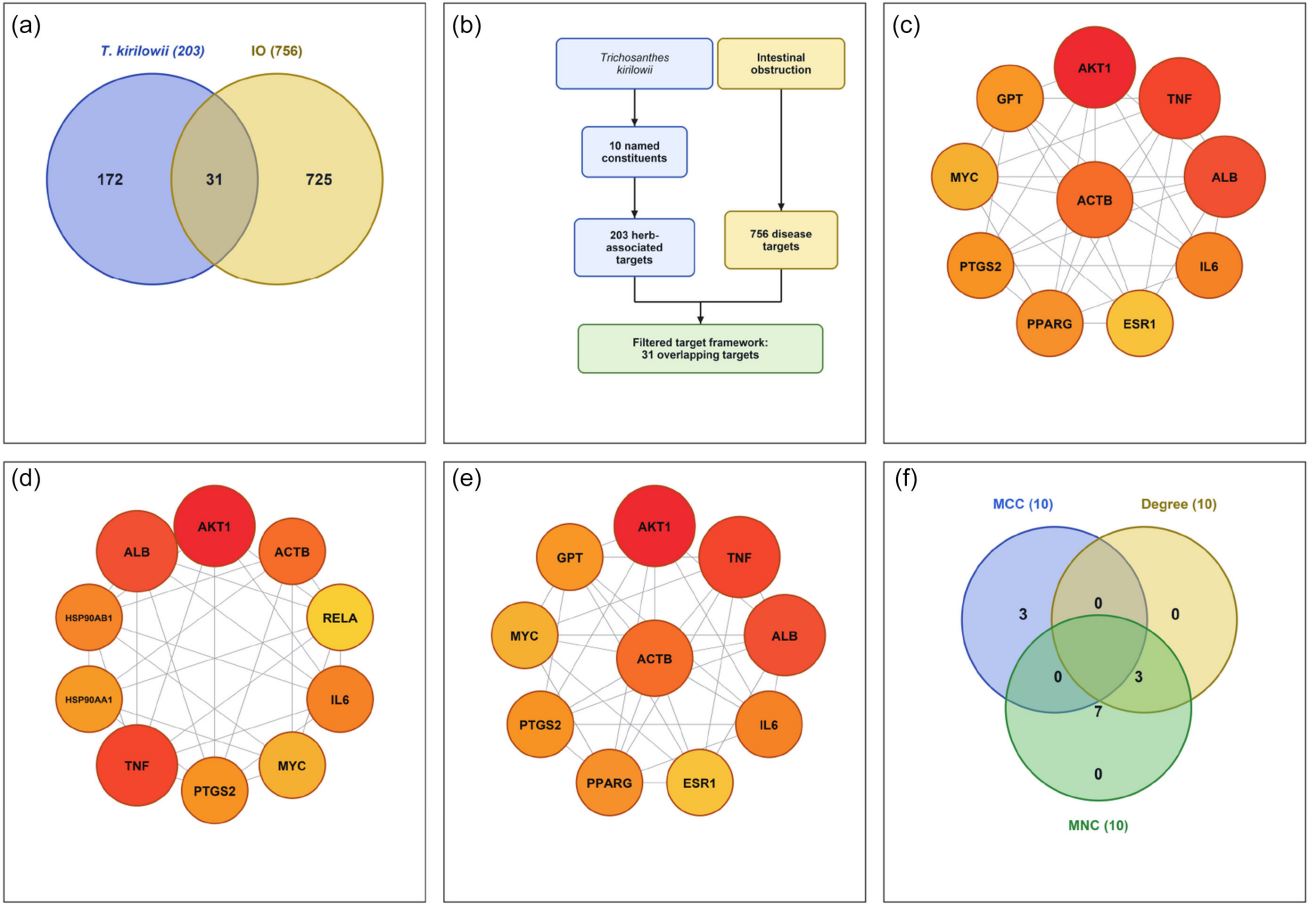
Filtered network‐pharmacology overview for *T. kirilowii* and intestinal obstruction. (a) Overlap between herb‐associated and intestinal‐obstruction targets. (b) Target‐source distribution from the herb‐ and disease‐target screening workflow. (c–e) CytoHubba‐based topological analysis of the filtered PPI network showing the top 10 hub genes identified by Degree (c), MCC (d), and MNC (e) algorithms. (f) Venn analysis showing the overlap among the three independent hub‐selection methods.

**TABLE 1:** Summary of target acquisition, overlap analysis, and STRING‐based PPI network construction for *T. kirilowii* and intestinal obstruction.

Quantity	Value
GeneCards primary filtered set (relevance ≥ 10)	698
OMIM additional unique genes	58
Final primary disease union	756
Broadened herb‐associated target set	203
Primary overlap	31
STRING mapped nodes	28
STRING edges	102
Defined human‐gene background	19,500
Primary hypergeometric *p*‐value	6.38 × 10^−11^

### 3.2 GO and KEGG Enrichment Analysis

The enrichment profile emphasized inflammation, survival signaling, cytoskeletal regulation, and arachidonic acid metabolism. GO terms included response to cytokine, regulation of apoptotic signaling, actin filament organization, and focal adhesion (Figure 2). KEGG terms included TNF signaling, PI3K‐Akt signaling, MAPK signaling, regulation of actin cytoskeleton, cytokine–cytokine receptor interaction, and arachidonic acid metabolism (Figure 3). Generic annotations such as pathways in cancer and viral infection pathways were retained but interpreted cautiously as multifunctional‐gene annotations (Table S18).

**FIGURE 2:**
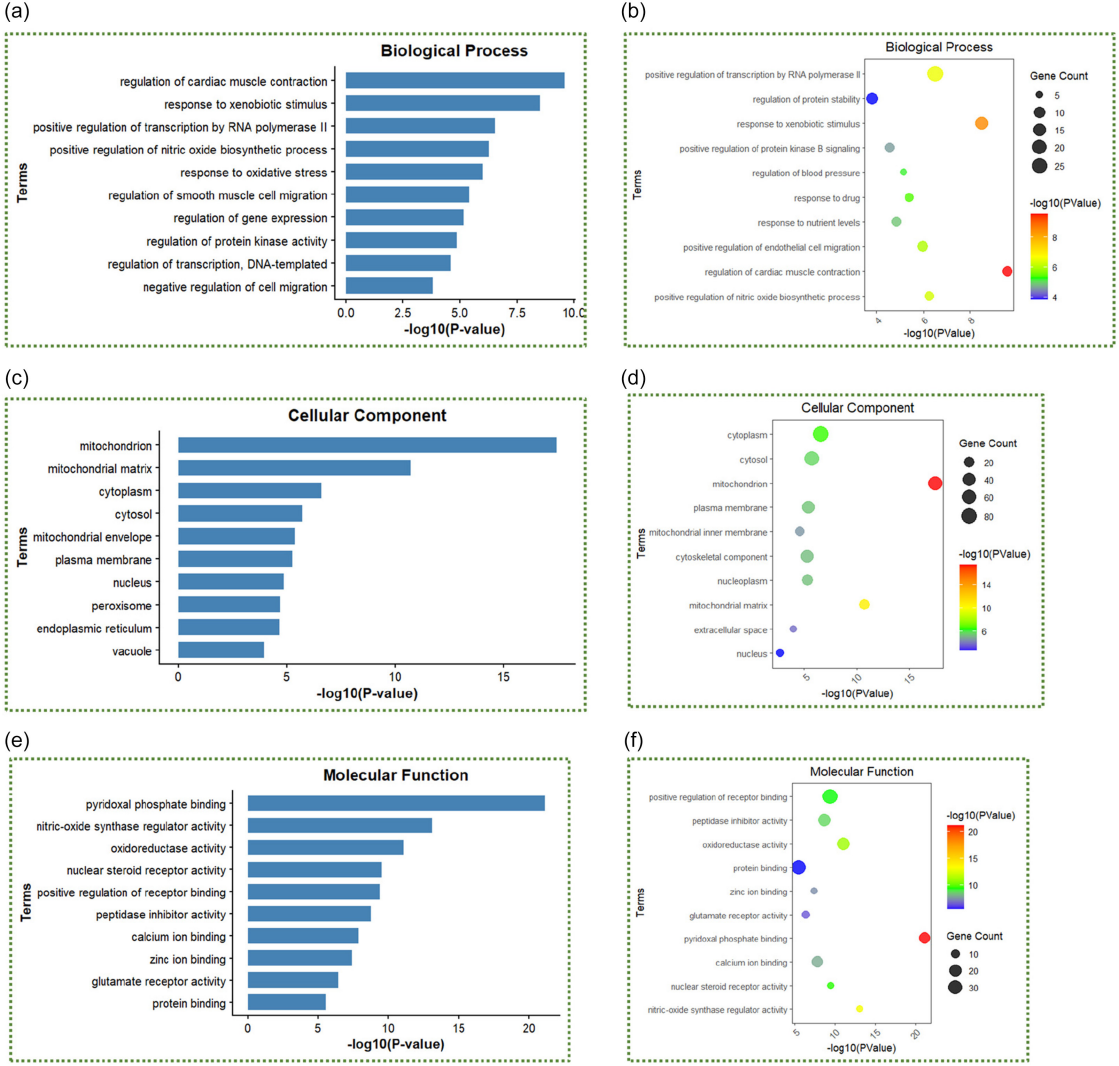
Gene Ontology (GO) enrichment analysis of the filtered *T*. *kirilowii*–intestinal obstruction overlap genes. (a) Bar plot of enriched biological process (BP) terms ranked by −log10(*p*‐value). (b) Bubble plot of enriched BP terms. (c) Bar plot of enriched cellular component (CC) terms ranked by −log10(*p*‐value). (d) Bubble plot of enriched CC terms. (e) Bar plot of enriched molecular function (MF) terms ranked by −log10(*p*‐value). (f) Bubble plot of enriched MF terms. In the bubble plots, bubble size indicates gene count and colour indicates −log10(*p*‐value).

**FIGURE 3:**
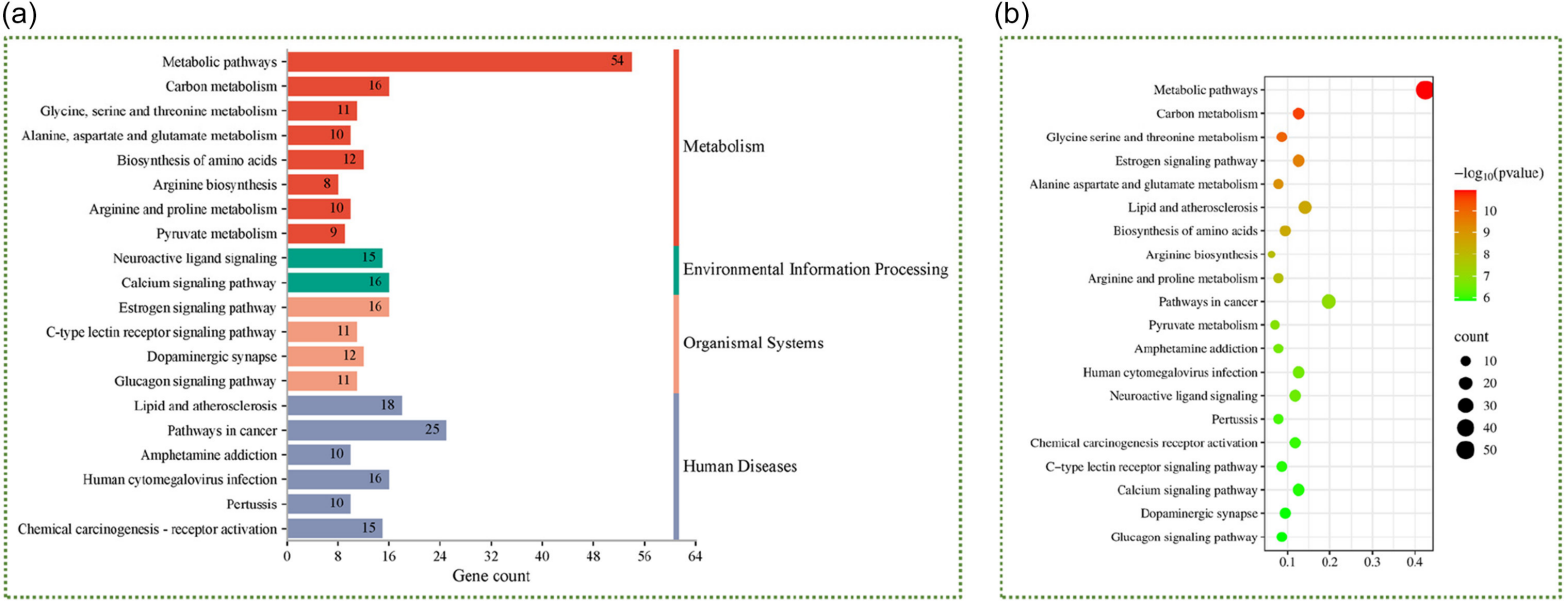
KEGG pathway enrichment analysis of the filtered overlap genes. (a) Bar plot of enriched KEGG pathways grouped into four categories: Metabolism, Environmental Information Processing, Organismal Systems, and Human Diseases; the number on each bar indicates the gene count. (b) Bubble plot of the same pathways, in which bubble size indicates gene count and colour indicates −log10(*p*‐value).

### 3.3 Expanded 10‐Compound Docking Panel

The expanded MOE docking screen evaluated ten named *T. kirilowii* constituents against seven targets (Figures 4 and 5; Tables 2 and S15). β‐D‐glucopyranosyl‐α‐spinasterol showed the strongest overall profile (mean score −8.19 kcal/mol; seven targets <= −7 kcal/‍mol; best target ALB, −9.97 kcal/mol) (Figures 6 and 7). Other favorable compounds included stigmast‐7‐en‐3beta‐ol 3‐O‐beta‐D‐glucopyranoside (mean −6.83), Cucurbitacin B (mean −6.79), Bryonolic acid (mean −6.73), and Cucurbitacin D (mean −6.62). Detailed residue–level interaction profiles for each docked complex are provided in Tables S1–S14.

**FIGURE 4:**
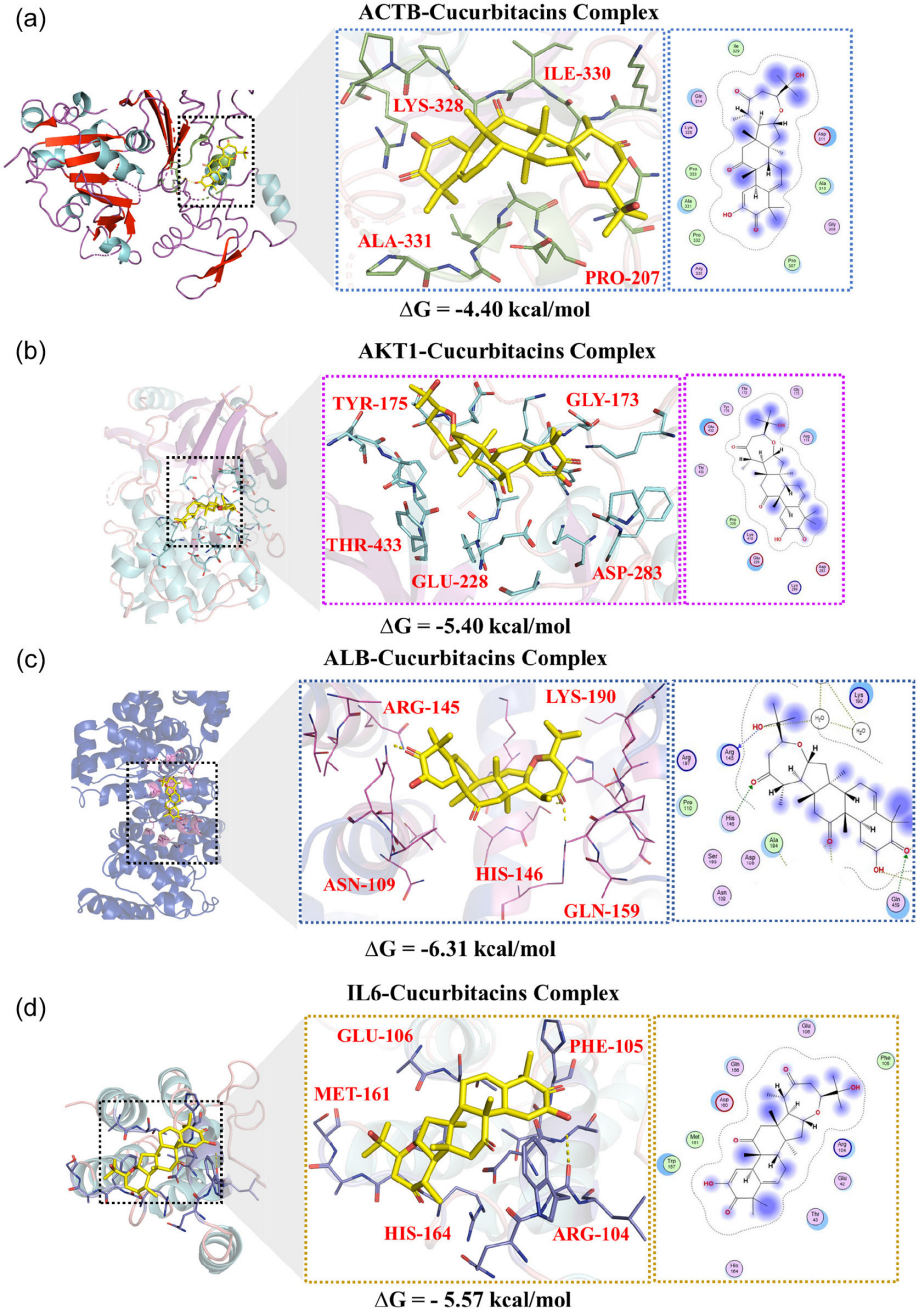
MOE docking interaction diagrams of Cucurbitacin E with four target proteins: (a) ACTB (ΔG = −4.40 kcal/mol), (b) AKT1 (ΔG = −5.40 kcal/mol), (c) ALB (ΔG = −6.31 kcal/mol), and (d) IL6 (ΔG = −5.57 kcal/mol). For each complex, the left image shows the overall protein structure with the binding site boxed, the middle image shows a close‐up 3D view of the docked ligand (yellow sticks) with key interacting residues labelled, and the right image shows the corresponding 2D ligand–interaction diagram.

**FIGURE 5:**
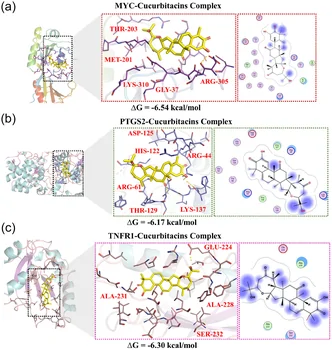
MOE docking interaction diagrams of Cucurbitacin E with three target proteins: (a) MYC (ΔG = −6.54 kcal/mol), (b) PTGS2 (ΔG = −6.17 kcal/mol), and (c) TNFR1/TNFRSF1A (ΔG = −6.30 ‍kcal/‍‍mol). For each complex, the left image shows the overall protein structure with the binding site boxed, the middle image shows a close‐up 3D view of the docked ligand (yellow sticks) with key interacting residues labelled, and the right image shows the corresponding 2D ligand–interaction diagram. For TNFR1/TNFRSF1A, residue labels are shown for pose visualization only and were not used for residue‐level interpretation.

**TABLE 2:** MOE docking analysis of 10 *T. kirilowii* constituents against intestinal‐obstruction‐associated targets, showing mean score, best target, best score, and number of high‐affinity interactions (≤−7.0 kcal/mol).

Compound	Class	Mean score	Best target	Best score	Targets <= −7
Cucurbitacin E	Cucurbitane triterpenoid	−5.77	MYC	−6.54	0
β‐D‐glucopyranosyl‐α‐spinasterol	Sterol glycoside	−8.19	ALB	−9.97	7
Cucurbitacin B	Cucurbitane triterpenoid	−6.79	PTGS2	−7.42	3
Cucurbitacin D	Cucurbitane triterpenoid	−6.62	MYC	−7.11	2
α‐Spinasterol	Phytosterol	−6.2	ALB	−7.76	1
Stigmast‐7‐en‐3β‐ol	Phytosterol	−5.69	ALB	−6.84	0
Stigmast‐7‐en‐3β‐ol 3‐O‐β‐D‐glucopyranoside	Sterol glycoside	−6.83	ALB	−8.11	2
Bryonolic acid	Pentacyclic triterpenoid	−6.73	PTGS2	−7.56	2
Karounidiol	Pentacyclic triterpenoid	−6.03	TNFR1	−6.52	0
Chrysoeriol	Flavonoid	−5.97	PTGS2	−7.22	2

**FIGURE 6:**
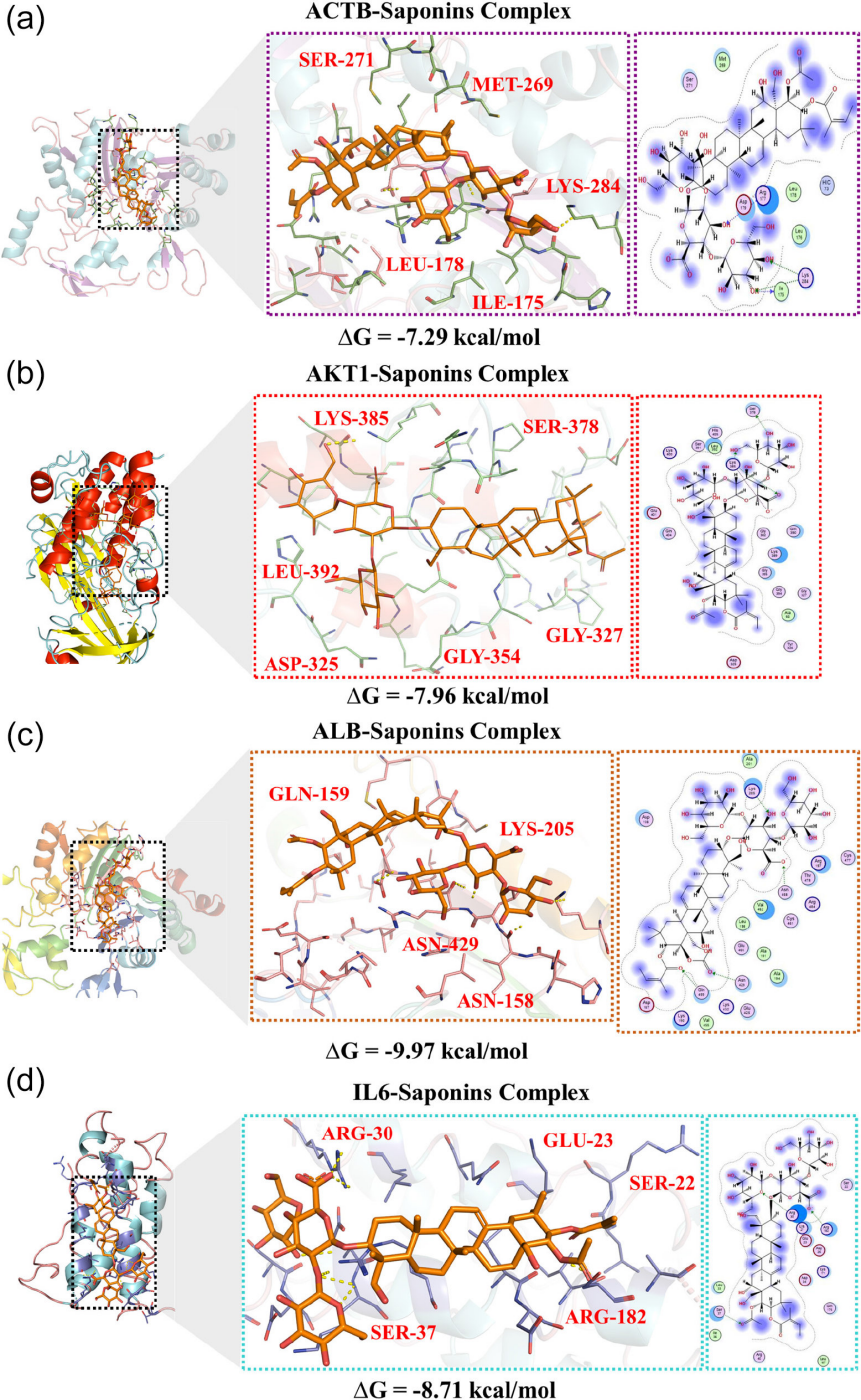
MOE docking interaction diagrams of β‐D‐glucopyranosyl‐α‐spinasterol with four target proteins: (a) ACTB (ΔG = −7.29 kcal/mol), (b) AKT1 (ΔG = −7.96 kcal/mol), (c) ALB (ΔG = −9.97 kcal/mol), and (d) IL6 (ΔG = −8.71 kcal/mol). For each complex, the left image shows the overall protein structure with the binding site boxed, the middle image shows a close‐up 3D view of the docked ligand (orange sticks) with key interacting residues labelled, and the right image shows the corresponding 2D ligand–interaction diagram.

**FIGURE 7:**
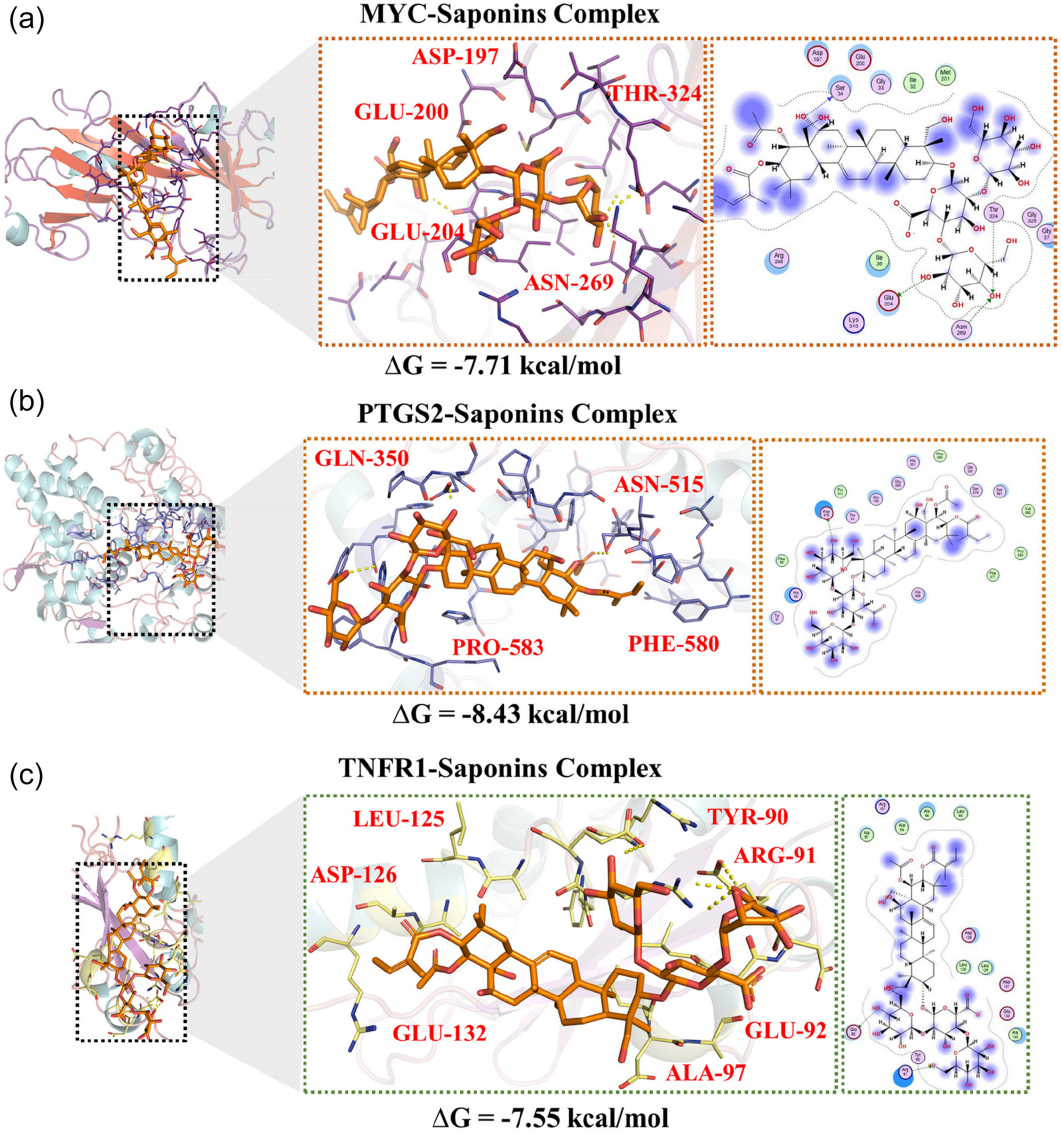
MOE docking interaction diagrams of β‐D‐glucopyranosyl‐α‐spinasterol with three target proteins: (a) MYC (ΔG = −7.71 kcal/mol), (b) PTGS2 (ΔG = −8.43 kcal/mol), and (c) TNFR1/TNFRSF1A (ΔG = −7.55 kcal/mol). For each complex, the left image shows the overall protein structure with the binding site boxed, the middle image shows a close‐up 3D view of the docked ligand (orange sticks) with key interacting residues labelled, and the right image shows the corresponding 2D ligand–interaction diagram. For TNFR1/TNFRSF1A, residue labels are shown for pose visualization only and were not used for residue‐level interpretation.

### 3.4 Docking Validation and MD/MM/GBSA Endpoint Analyses

Native‐ligand or site‐specific redocking passed for ACTB, AKT1, ALB, PTGS2, and TNFR1/TNFRSF1A, with RMSD values from 0.94 to 1.78 Å; IL6 and MYC were handled as alternative‐site validation cases (Table S16). For the Cucurbitacin E and β‐‍D‐glucopyranosyl‐α‐spinasterol systems, full 0–100 ns trajectories were visualized for RMSD, Rg, and SASA (Figures 8 and 9). The equilibrated 60–100 ns segment was used for hydrogen‐bond occupancy, MM/GBSA endpoint binding‐energy estimation, component energies, and per‐residue energy decomposition, where a reliable export was available (Tables 3 and S19–S22). TNFR1 interpretation excludes unverified residue‐contact identities.

**FIGURE 8:**
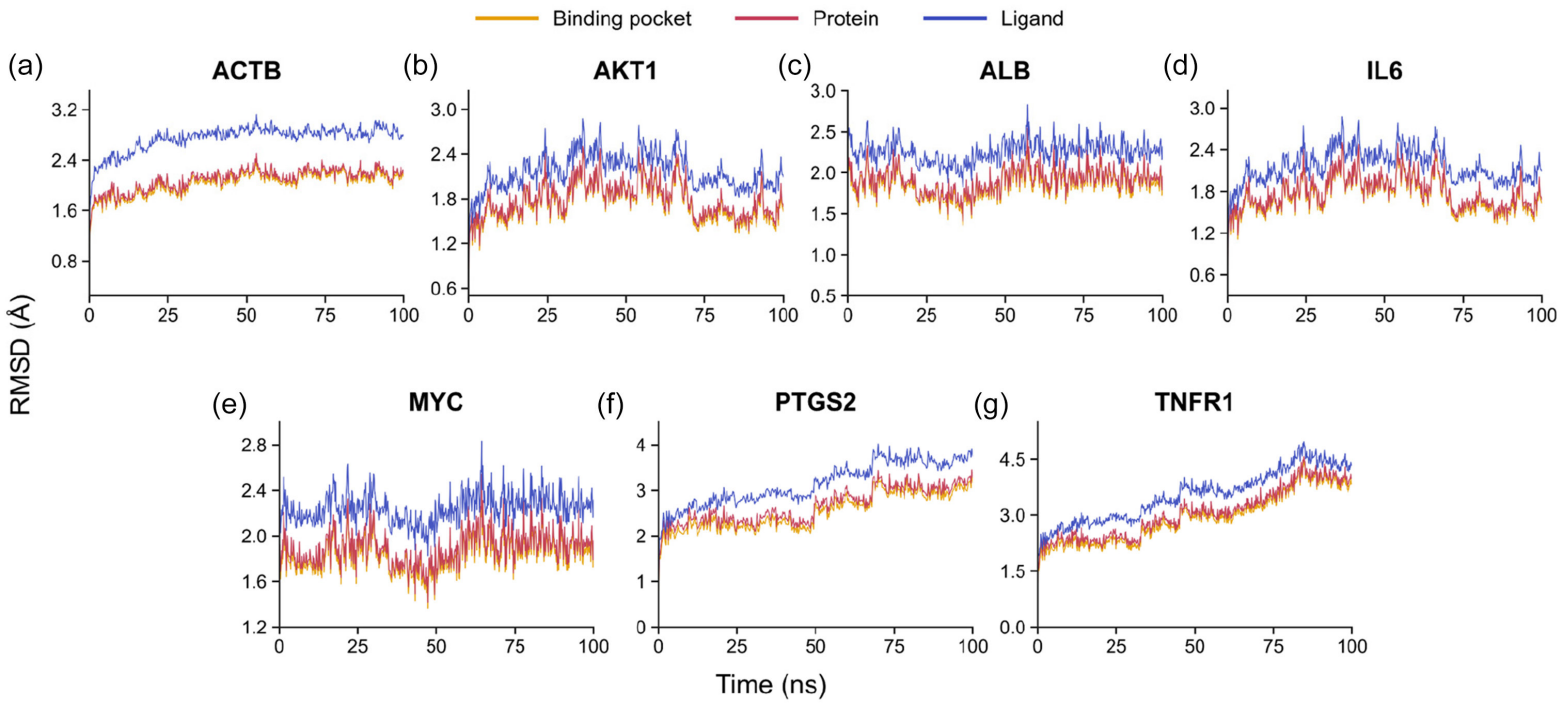
RMSD trajectories of Cucurbitacin E–target complexes during 100 ns MOE molecular dynamics simulations: (a) ACTB, (b) AKT1, (c) ALB, (d) IL6, (e) MYC, (f) PTGS2, and (g) TNFR1/TNFRSF1A. Binding‐pocket, protein‐backbone, and ligand RMSD values are plotted against simulation time. For each panel, the first and last configurations displayed in the original origin graph were mapped linearly to 0 and 100 ns, respectively; the original RMSD values were retained without smoothing or interpolation.

**FIGURE 9:**
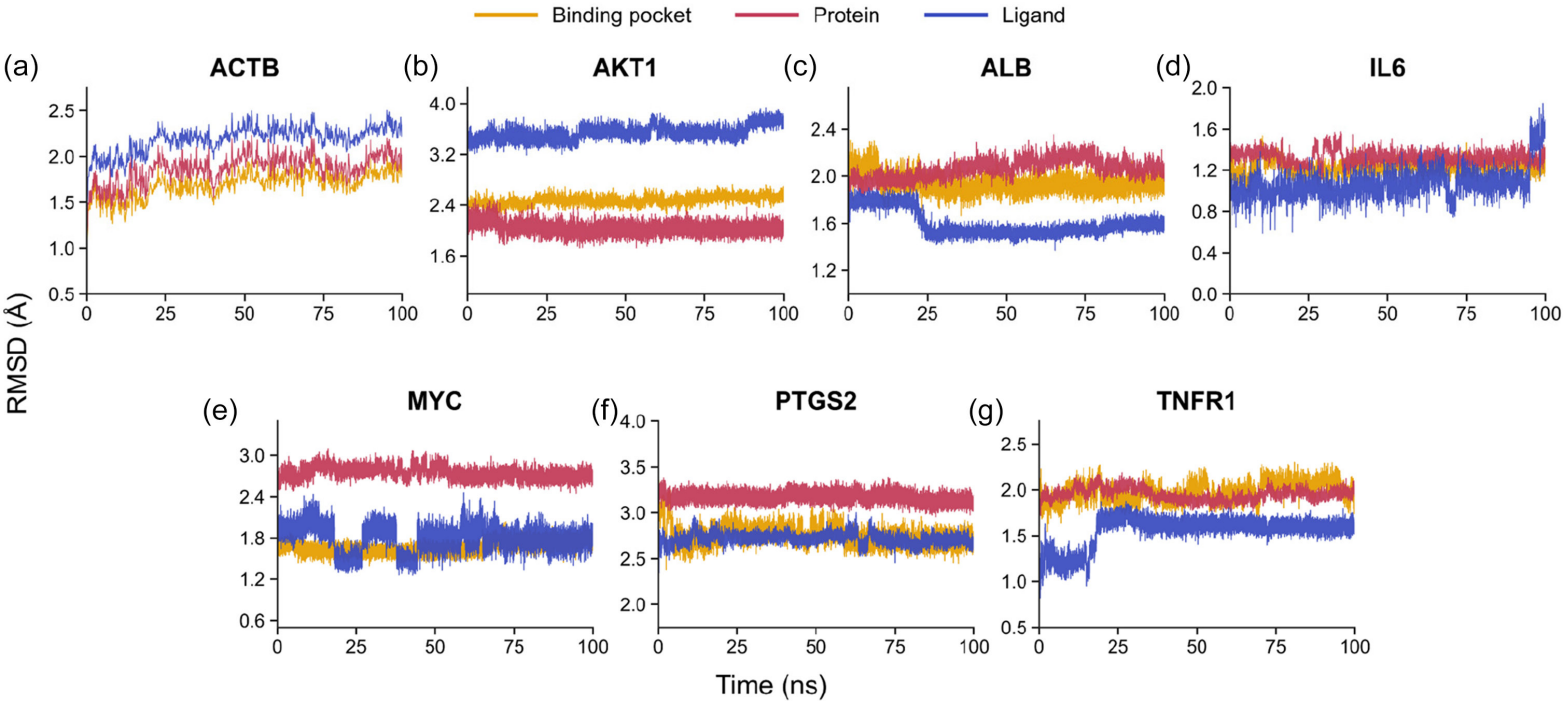
RMSD trajectories of β‐D‐glucopyranosyl‐α‐spinasterol‐target complexes during 100 ns MOE molecular dynamics simulations: (a) ACTB, (b) AKT1, (c) ALB, (d) IL6, (e) MYC, (f) PTGS2, and (g) TNFR1/TNFRSF1A. Binding‐pocket, protein‐backbone, and ligand RMSD values are plotted against simulation time. For each panel, the first and last configurations displayed in the original origin graph were mapped linearly to 0 and 100 ns, respectively; the original RMSD values were retained without smoothing or interpolation.

**TABLE 3:** Docking and MD analysis of Cucurbitacin E and β‐D‐glucopyranosyl‐α‐spinasterol complexes with intestinal‐obstruction‐related targets.

Target	Ligand	Docking score	Ligand RMSD, Å	Rg, Å	Mean H‐bond occupancy, %	MM/GBSA endpoint binding‐energy estimate, kcal/mol
ACTB	Cucurbitacin E	−4.4	2.1 ± 0.25	20.77 ± 0.14	33.8	−31.3 ± 5.7
AKT1	Cucurbitacin E	−5.4	1.96 ± 0.23	22.43 ± 0.16	46.7	−40.8 ± 5.8
ALB	Cucurbitacin E	−6.31	1.87 ± 0.19	27.25 ± 0.12	26	−36.3 ± 6
IL6	Cucurbitacin E	−5.57	2.02 ± 0.22	15.55 ± 0.1	41.3	−34.3 ± 6.1
MYC	Cucurbitacin E	−6.54	2.04 ± 0.26	18.74 ± 0.14	25.6	−48.5 ± 6
PTGS2	Cucurbitacin E	−6.17	2.16 ± 0.38	24.65 ± 0.2	22.2	−37.6 ± 6.2
TNFR1	Cucurbitacin E	−5.98	2.17 ± 0.37	18.15 ± 0.2	18.4	−33.1 ± 6.4
ACTB	β‐D‐glucopyranosyl‐α‐spinasterol	−7.29	2.09 ± 0.3	20.85 ± 0.12	37.1	−38.6 ± 5.9
AKT1	β‐D‐glucopyranosyl‐α‐spinasterol	−7.96	2.79 ± 0.4	22.64 ± 0.23	19.8	−35.9 ± 6.3
ALB	β‐D‐glucopyranosyl‐α‐spinasterol	−9.97	2.04 ± 0.25	27.16 ± 0.11	42.4	−55.9 ± 5.6
IL6	β‐D‐glucopyranosyl‐α‐spinasterol	−8.71	1.98 ± 0.19	15.55 ± 0.09	45.4	−48.6 ± 5.9
MYC	β‐D‐glucopyranosyl‐α‐spinasterol	−7.71	2.47 ± 0.34	18.92 ± 0.16	27.7	−40.4 ± 5.8
PTGS2	β‐D‐glucopyranosyl‐α‐spinasterol	−8.43	3.06 ± 0.47	24.85 ± 0.25	11	−31.2 ± 6
TNFR1	β‐D‐glucopyranosyl‐α‐spinasterol	−7.24	2.32 ± 0.29	18.15 ± 0.16	34	−44 ± 5.7

The full 0–100 ns Rg and SASA trajectories provided an orthogonal check on the RMSD‐based stability assessment (Figures S1 and S2). Across the fourteen lead ligand–target simulations, the Rg profiles showed an early relaxation phase followed by bounded fluctuations rather than progressive expansion, indicating that the protein folds remained globally compact after complex formation. This behavior was most consistent with equilibrated conformational sampling rather than ligand‐induced unfolding, and it supports the interpretation that the docking‐derived complexes were structurally persistent during the 100 ns trajectories.

The SASA profiles showed the same qualitative pattern: after the initial adjustment of solvent‐exposed surface area, each complex fluctuated within a narrow target‐specific envelope without sustained drift. Because SASA is sensitive to exposure of hydrophobic and polar surface patches, the absence of monotonic surface expansion suggests that neither Cucurbitacin E nor β‐D‐glucopyranosyl‐α‐spinasterol caused broad destabilization of the simulated target structures. Taken together with RMSD, hydrogen‐bond persistence, and MM/GBSA endpoint binding‐energy estimates, these Rg/SASA data strengthen the conclusion that the two lead compounds form dynamically stable, target‐dependent complexes under the standardized 300 K simulation condition.

## 4 Discussion

The integrated workflow combines a 10‐compound named docking panel, a GeneCards relevance threshold for disease‐target prioritization, statistical overlap enrichment, GO/KEGG analysis of the filtered target set, explicit hub/biological target‐selection logic, MOE docking validation where appropriate controls were available, and MD/MM/GBSA endpoint binding‐energy estimation with residue‐level evidence only where supported by verified exports. For TNFR1, conclusions are limited to the verified 9S3J/A1JLJ redocking result, harmonized docking scores, hydrogen‐bond persistence, and complex‐level/component energetics.

The filtered target workflow reduced the disease union from 11,207 raw GeneCards hits to a 756‐gene primary disease set. The resulting 31‐target overlap was statistically significant and produced a compact STRING network of 28 mapped nodes and 102 edges, making the network pharmacology interpretation less vulnerable to the near‐certain overlap problem caused by an unfiltered disease list.

The expanded docking panel broadened the structural basis for compound prioritization. β‐D‐glucopyranosyl‐α‐spinasterol remained the strongest current ligand, but Cucurbitacin B, Cucurbitacin D, stigmast‐7‐en‐3beta‐ol 3‐O‐beta‐D‐glucopyranoside, Bryonolic acid, and Chrysoeriol also showed target‐selective favorable docking, especially toward ALB, IL6, MYC, and PTGS2.

The MD/MM/GBSA analyses provide additional stability and endpoint‐energy support for the two lead ligands. The 300 K trajectory temperature should be regarded as a standardized computational condition, not a direct physiological‐temperature simulation; confirmation at 310 K and in biochemical or cellular systems would strengthen translational interpretation. These computational findings remain hypothesis‐generating and should be followed by biochemical target‐engagement assays, cellular validation, and disease‐relevant experimental models before biological or clinical claims are made.

## 5 Conclusion

This study combines filtered network pharmacology, an expanded 10‐compound MOE docking panel, docking validation, and 100 ns MD/MM/GBSA analyses to clarify how *T. kirilowii* bioactives may act against intestinal obstruction‐related pathways. The analysis identified a statistically enriched 31‐target herb–disease overlap, a compact STRING network, disease‐relevant TNF/PI3K‐Akt/MAPK/actin/arachidonic‐acid pathway annotations, a transparent hub‐plus‐biological target‐selection rationale, and target‐selective docking patterns across 10 named constituents. β‐‍D‐glucopyranosyl‐α‐spinasterol showed the strongest overall docking profile, while several additional constituents provided broader support for plant‐level chemical diversity. The findings support a multicompound, multitarget computational hypothesis for experimental validation.

## Author Contributions

**Tianying Wu:** conceptualization; methodology; writing – original draft. **Xiaofen Lin:** data curation; formal analysis; software; visualization. **Wencong Lin:** investigation, methodology, and data curation. **Lixia Huang:** formal analysis; investigation; validation; visualization. **Yuewu Lin:** conceptualization; project administration; resources; supervision; writing – review & editing. All authors read and approved of the final manuscript.

## Funding

The authors have no report.

## Ethics Approval and Consent to Participate

Not applicable. This study did not involve human participants, human tissue, or animal subjects. All analyses were performed using publicly available databases and computational simulations.

## Conflicts of Interest

The authors declare no conflict of interest.

## Supporting information

Supplementary Material

## Data Availability

The processed network/enrichment tables, docking poses, input structures, parameter files, and trajectory‐derived analysis tables are available from the corresponding author on reasonable demand.
