# Supplementary material for: Elucidating the MultiPathway Therapeutic Potential of Trichosanthes kirilowii Bioactives in Intestinal Obstruction via Network Pharmacology and Molecular Dynamics
Source: ChemistryOpen. 2026 Oct 5;15(10):e70312. doi: 10.1002/open.70312 (PMC13639117; doi:10.1002/open.70312)
Supplement: Supplementary file 1 [file OPEN-15-e70312-s001.pdf]

Supplementary Material

Elucidating the Multi-Pathway Therapeutic Potential of *Trichosanthes kirilowii* Bioactives in Intestinal Obstruction via Network Pharmacology and Molecular Dynamics

Tianying Wu[1+], Xiaofen Lin[1+], Wencong Lin[2+], Lixia Huang[3], Yuewu Lin[1*]

[1]Department of Geriatrics, Zhangzhou Affiliated Hospital of Fujian Medical University, Zhangzhou 363000, China

[2]Department of Oncology, Zhangzhou Affiliated Hospital of Fujian Medical University, Zhangzhou 363000, China

[3]Department of Nuclear Medicine, Zhangzhou Affiliated Hospital of Fujian Medical University, Zhangzhou 363000, China

[+] These authors contributed equally to this work (co-first authors).

[*] **Corresponding author:** Yuewu Lin

Email: Yuewulin1@outlook.com

Table S1. Interaction analysis between ACTB and Cucurbitacin E.

Drug	Protein	Docking Score MOE docking score (kcal/mol equivalent)	Amino Acids	Interaction Category
Cucurbitacin E	ACTB	-4.40	ILE-329	Hydrophobic
			GLN-314	Hydrophobic
			LYS-328	Hydrophobic
			PRO-333	Hydrophobic
			ALA-331	Hydrophobic
			PRO-332	Hydrophobic
			ARG-335	Hydrophobic
Cucurbitacin E			PRO-307	

Drug	Protein	Docking Score MOE docking score (kcal/mol equivalent)	Amino Acids	Interaction Category
Cucurbitacin E			GLY-308	
Cucurbitacin E			ALA-310	
Cucurbitacin E			ASP311	

Table S2. Interaction analysis between AKT1 and Cucurbitacin E.

Drug	Protein	Docking Score MOE docking score (kcal/mol equivalent)	Amino Acids	Interaction Category
Cucurbitacin E			THR-172	Hydrophobic
			TYR-175	Hydrophobic
			GLU-432	Hydrophobic
	AKT1	-5.40	THR-433	Hydrophobic
			PRO-208	Hydrophobic
			LYS-419	Hydrophobic
			GLU-228	Hydrophobic
Cucurbitacin E			LYS-289	Hydrophobic
Cucurbitacin E			ASP-283	Hydrophobic
Cucurbitacin E			ARG-174	Hydrophobic

Table S3. Interaction analysis between ALB and Cucurbitacin E.

Drug	Protein	Docking Score MOE docking score (kcal/mol equivalent)	Amino Acids	Interaction Category
Cucurbitacin E	ALB		ARG-145	Hydrophobic
			ARG-197	Hydrophobic
			HIS-146	Hydrogen Bond
			SER-193	Hydrophobic
		-6.31	ASN-109	Hydrophobic
			ALA-194	Hydrophobic
			GLN-459	Hydrophobic
			LYS-190	Hydrophobic

Table S4. Interaction analysis between IL6 and Cucurbitacin E.

Drug	Protein	Docking Score MOE docking score (kcal/mol equivalent)	Amino Acids	Interaction Category
Cucurbitacin E	IL6		GLN-156	Hydrophobic
			ASP-160	Hydrophobic
			MET-161	Hydrophobic
			TRP-157	Hydrophobic
		-5.57	HIS-164	Hydrophobic
			THR-43	Hydrophobic
			GLU-42	Hydrophobic
			ARG-104	Hydrophobic
			PHE-105	Hydrophobic

Table S5. Interaction analysis between MYC and Cucurbitacin E.

Drug	Protein	Docking Score MOE docking score (kcal/mol equivalent)	Amino Acids	Interaction Category
Cucurbitacin E	MYC		ASP-197	Hydrophobic
			GLY-33	Hydrophobic
			GLU-200	Hydrophobic
			THR-203	Hydrophobic
		-6.54	GLY-325	Hydrophobic
			MET-201	Hydrophobic
			THR-324	Hydrophobic
			GLU-204	Hydrophobic
			THR-311	Hydrophobic
Cucurbitacin E			ASN-269	Hydrophobic
Cucurbitacin E			THR-312	Hydrophobic
Cucurbitacin E			LYS-310	Hydrophobic
Cucurbitacin E			ILE-36	Hydrophobic
Cucurbitacin E			GLY-37	Hydrophobic
Cucurbitacin E			ARG-305	Hydrophobic
Cucurbitacin E			ILE-306	Hydrophobic

Table S6. Interaction analysis between PTGS2 and Cucurbitacin E.

Drug	Protein	Docking Score MOE docking score (kcal/mol equivalent)	Amino Acids	Interaction Category
Cucurbitacin E	PTGS2		HIS-122	Hydrophobic
			SER-126	Hydrophobic
			THR-62	Hydrophobic
			THR-60	Hydrophobic
		-6.17	ARG-61	Hydrophobic
			PRO-128	Hydrophobic
			THR-129	Hydrophobic
			LYS-137	Hydrophobic
			GLN-358	Hydrophobic
Cucurbitacin E			ARG-44	Hydrophobic
Cucurbitacin E			PRO127	Hydrophobic

Table S7. Interaction analysis between TNFR1/TNFRSF1A and Cucurbitacin E.

Drug	Protein	Docking Score MOE docking score (kcal/mol equivalent)	Amino Acids	Interaction Category
Cucurbitacin E	TNFR1/TNFRSF1A	-5.98	Not reported: a verified residue-contact export for the final 9S3J pose was unavailable.	Not assigned

Table S8. Interaction analysis between ACTB and β-D-glucopyranosyl-α-spinasterol.

Drug	Protein	Docking Score MOE docking score (kcal/mol equivalent)	Amino Acids	Interaction Category
β-D-glucopyranosyl-α-spinasterol	ACTB		MET-269	Hydrophobic
			SER-271	Hydrophobic
			ASP-179	Hydrophobic
			ARG-177	Hydrophobic
		-7.29	LEU-178	Hydrophobic
			LEU-176	Hydrophobic
			LYS-284	Hydrogen Bond
			ILE-175	Hydrogen Bond

Table S9. Interaction analysis between AKT1 and β-D-glucopyranosyl-α-spinasterol.

Drug	Protein	Docking Score MOE docking score (kcal/mol equivalent)	Amino Acids	Interaction Category
β-D-glucopyranosyl-α-spinasterol	AKT1		SER-378	Hydrogen Bond
			LYS-385	Hydrogen Bond
			LEU-392	Hydrophobic
			SER-361	Hydrophobic
		-7.96	LYS-400	Hydrophobic
			GLU-401	Hydrophobic
			GLN-404	Hydrophobic
			ALA-50	Hydrophobic
			GLY-394	Hydrophobic

Table S10. Interaction analysis between ALB and β-D-glucopyranosyl-α-spinasterol.

Drug	Protein	Docking Score MOE docking score (kcal/mol equivalent)	Amino Acids	Interaction Category
β-D-glucopyranosyl-α-spinasterol	ALB	-9.97	ASP-108	Hydrophobic
			ALA-201	Hydrophobic
			LYS-205	Hydrogen Bond
			ARG-197	Hydrophobic
			THR-478	Hydrophobic
			ASN-458	Hydrogen Bond
			ARG-484	Hydrophobic
			CYS-451	Hydrophobic
			VAL-462	Hydrophobic
β-D-glucopyranosyl-α-spinasterol			ASN-429	Hydrogen Bond
β-D-glucopyranosyl-α-spinasterol			GLN-459	Hydrogen Bond
β-D-glucopyranosyl-α-spinasterol			ASP-187	Hydrophobic
β-D-glucopyranosyl-α-spinasterol			LYS-190	Hydrophobic
β-D-glucopyranosyl-α-spinasterol			VAL-455	Hydrophobic

Drug	Protein	Docking Score MOE docking score (kcal/mol equivalent)	Amino Acids	Interaction Category
β-D-glucopyranosyl-α-spinasterol			LYS-432	Hydrophobic
β-D-glucopyranosyl-α-spinasterol			GLU-425	Hydrophobic
β-D-glucopyranosyl-α-spinasterol			ALA-194	Hydrophobic
β-D-glucopyranosyl-α-spinasterol			ALA-191	Hydrophobic

Table S11. Interaction analysis between IL6 and β-D-glucopyranosyl-α-spinasterol.

Drug	Protein	Docking Score MOE docking score (kcal/mol equivalent)	Amino Acids	Interaction Category
β-D-glucopyranosyl-α-spinasterol	IL6		SER-22	Hydrophobic
			ARG-182	Hydrogen Bond
			ARG-30	Hydrogen Bond
			LYS-27	Hydrophobic
		-8.71	GLU-23	Hydrophobic
			ASP-26	Hydrophobic
			LYS-171	Hydrophobic
			ASP-34	Hydrophobic

Drug	Protein	Docking Score MOE docking score (kcal/mol equivalent)	Amino Acids	Interaction Category
			GLN-178	Hydrophobic
β-D-glucopyranosyl-α-spinasterol			LEU-167	Hydrophobic
β-D-glucopyranosyl-α-spinasterol			ARG-40	Hydrophobic
β-D-glucopyranosyl-α-spinasterol			ILE-36	Hydrophobic
β-D-glucopyranosyl-α-spinasterol			SER-37	Hydrophobic
β-D-glucopyranosyl-α-spinasterol			LEU-33	Hydrophobic

Table S12. Interaction analysis between MYC and β-D-glucopyranosyl-α-spinasterol.

Drug	Protein	Docking Score MOE docking score (kcal/mol equivalent)	Amino Acids	Interaction Category
β-D-glucopyranosyl-α-spinasterol	MYC		ASP-197	Hydrophobic
			SER-34	Hydrophobic
			GLU-200	Hydrophobic
			GLY-33	Hydrophobic

**Drug**	**Protein**	**Docking Score MOE docking score (kcal/mol equivalent)**	**Amino Acids**	**Interaction Category**
		-7.71	ILE-32	Hydrophobic
			MET-201	Hydrophobic
			ARG-298	Hydrophobic
			ILE-36	Hydrophobic
			GLU-204	Hydrogen Bond
β-D-glucopyranosyl-α-spinasterol			LYS-310	Hydrophobic
β-D-glucopyranosyl-α-spinasterol			ASN-269	Hydrogen Bond
β-D-glucopyranosyl-α-spinasterol			GLY-37	Hydrophobic
β-D-glucopyranosyl-α-spinasterol			GLY-325	Hydrophobic
β-D-glucopyranosyl-α-spinasterol			THR-324	Hydrogen Bond

Table S13. Interaction analysis between PTGS2 and β-D-glucopyranosyl-α-spinasterol.

Drug	Protein	Docking Score MOE docking score (kcal/mol equivalent)	Amino Acids	Interaction Category
β-D-glucopyranosyl-α-spinasterol	PTGS2	-8.43	ASP-515	Hydrogen Bond
			PRO-514	Hydrophobic
			THR-94	Hydrophobic
			GLN-192	Hydrophobic
			GLY-354	Hydrophobic
			HIS-351	Hydrophobic
			PHE-580	Hydrophobic
			GLN-350	Hydrophobic
			SER-579	Hydrophobic
β-D-glucopyranosyl-α-spinasterol			SER-581	Hydrophobic
β-D-glucopyranosyl-α-spinasterol			VAL-582	Hydrophobic
β-D-glucopyranosyl-α-spinasterol			PRO-583	Hydrophobic
β-D-glucopyranosyl-α-spinasterol			PHE-577	Hydrophobic
β-D-glucopyranosyl-α-spinasterol			HIS-356	Hydrophobic

Table S14. Interaction analysis between TNFR1/TNFRSF1A and β-D-glucopyranosyl-α-spinasterol.

**Drug**	**Protein**	**Docking Score MOE docking score (kcal/mol equivalent)**	**Amino Acids**	**Interaction Category**
β-D-glucopyranosyl-α-spinasterol	TNFR1/TNFRSF1A	-7.24	Not reported: a verified residue-contact export for the final 9S3J pose was unavailable.	Not assigned

Table S15. Expanded 10-compound MOE docking panel.

Code	Compound	Class	ACTB	AKT1	ALB	IL6	MYC	PTGS2	TNFR1/TNFRSF1A	Mean score	Best target	Best score	Targets ≤ -7
C01	Cucurbitacin E	Cucurbitane triterpenoid	-4.4	-5.4	-6.31	-5.57	-6.54	-6.17	-5.98	-5.77	MYC	-6.54	0

Code	Compound	Class	ACTB	AKT1	ALB	IL6	MYC	PTGS2	TNFR1/TNFRSF1A	Mean score	Best target	Best score	Targets ≤ -7
C02	β-D-glucopyranosyl-α-spinasterol	Sterol glycoside	-7.29	-7.96	-9.97	-8.71	-7.71	-8.43	-7.24	-8.19	ALB	-9.97	7
C03	Cucurbitacin B	Cucurbitane triterpenoid	-5.82	-7.18	-7.34	-6.88	-6.61	-7.42	-6.27	-6.79	PTGS2	-7.42	3
C04	Cucurbitacin D	Cucurbitane triterpenoid	-6.21	-6.37	-7.02	-6.64	-7.11	-6.93	-6.08	-6.62	MYC	-7.11	2
C05	α-Spinasterol	Phytosterol	-5.18	-6.44	-7.76	-6.31	-5.84	-6.15	-5.72	-6.2	ALB	-7.76	1
C06	Stigmast-7-en-3β-ol	Phytosterol	-4.87	-5.96	-6.84	-5.54	-5.49	-5.91	-5.21	-5.69	ALB	-6.84	0

Code	Compound	Class	ACTB	AKT1	ALB	IL6	MYC	PTGS2	TNFR1/TNFRSF1A	Mean score	Best target	Best score	Targets ≤ -7
C07	Stigmast-7-en-3β-ol 3-O-β-D-glucopyranoside	Sterol glycoside	-6.36	-6.92	-8.11	-7.26	-6.42	-6.58	-6.14	-6.83	ALB	-8.11	2
C08	Bryonolic acid	Pentacyclic triterpenoid	-5.74	-6.83	-6.47	-7.28	-6.31	-7.56	-6.89	-6.73	PTGS2	-7.56	2
C09	Karounidiol	Pentacyclic triterpenoid	-5.43	-6.11	-6.03	-5.77	-5.94	-6.41	-6.52	-6.03	TNFR1/TNFRSF1A	-6.52	0
C10	Chrysoeriol	Flavonoid	-4.63	-6.06	-5.28	-7.03	-5.44	-7.22	-6.11	-5.97	PTGS2	-7.22	2

Table S16. Docking validation by native-ligand or site-specific redocking.

Target	Structure	Reference ligand/control	Validation approach	RMSD (Å)	Status
ACTB	6NBW	Latrunculin B	Native-ligand redocking in the resolved Latrunculin B pocket	1.78	Pass
AKT1	4EJN	0R4	Native-ligand redocking	0.94	Pass
ALB	1E7F	Lauric acid (DAO)	Site-specific redocking in the crystallographic lauric-acid pocket	1.49	Pass
IL6	4NI9	SOMAmer-bound structure	Alternative-site validation		N/A
MYC	6E16	Protein-interface complex	Alternative-site validation		N/A
PTGS2	5F19	COX-2 active-site reference pose	Site-specific pose benchmark in the resolved 5F19 pocket	1.27	Pass
TNFR1/TNFRSF1A	9S3J	A1JLJ	Native-ligand redocking in 9S3J	1.12	Pass

Table S16A. MOE docking-site definition and verified contact-reporting status for docking reproducibility.

Target	Structure	MOE site definition	Verified site/contact reporting from final poses	Validation note
ACTB	6NBW	Resolved ligand region/Site Finder pocket converted to alpha-sphere dummy atoms	Cucurbitacin E: ILE329, GLN314, LYS328, PRO333, ALA331, PRO332, ARG335; β-D-glucopyranosyl-α-spinasterol: MET269, SER271, ASP179, ARG177, LEU178, LEU176, LYS284, ILE175	Redocking control: Latrunculin B, RMSD 1.78 Å
AKT1	4EJN	Resolved ligand region/Site Finder pocket converted to alpha-sphere dummy atoms	Cucurbitacin E: THR172, TYR175, GLU432, THR433, PRO208,	Redocking control: 0R4, RMSD 0.94 Å

Target	Structure	MOE site definition	Verified site/contact reporting from final poses	Validation note
			LYS419, GLU228, LYS289, ASP283, ARG174; β-D-glucopyranosyl-α-spinasterol: SER378, LYS385, LEU392, SER361, LYS400, GLU401, GLN404, ALA50, GLY394	
ALB	1E7F	Site-matched fatty-acid/albumin pocket and Site Finder pocket	Cucurbitacin E: ARG145, ARG197, HIS146, SER193, ASN109, ALA194, GLN459, LYS190; β-D-glucopyranosyl-α-spinasterol:	Site-specific redocking: lauric acid (DAO), RMSD 1.49 Å

Target	Structure	MOE site definition	Verified site/contact reporting from final poses	Validation note
			ASP108, ALA201, LYS205, ARG197, THR478, ASN458, ARG484, CYS451, VAL462, ASN429, GLN459	
IL6	4NI9	MOE Site Finder alternative pocket because no directly comparable small-molecule native control was available	Cucurbitacin E: GLN156, ASP160, MET161, TRP157, HIS164, THR43, GLU42, ARG104, PHE105; β-D-glucopyranosyl-α-spinasterol: SER22, ARG182, ARG30, LYS27, GLU23, ASP26, LYS171,	Alternative-site validation case

Target	Structure	MOE site definition	Verified site/contact reporting from final poses	Validation note
			ASP34, GLN178	
MYC	6E16	MOE Site Finder alternative pocket because no directly comparable small-molecule native control was available	Cucurbitacin E: ASP197, GLY33, GLU200, THR203, GLY325, MET201, THR324, GLU204, THR311, ASN269, LYS310; β-D-glucopyranosyl-α-spinasterol: ASP197, SER34, GLU200, GLY33, ILE32, MET201, ARG298, ILE36, GLU204, ASN269, THR324	Alternative-site validation case

Target	Structure	MOE site definition	Verified site/contact reporting from final poses	Validation note
PTGS2	5F19	Resolved COX-2 active-site region in 5F19 and the corresponding MOE Site Finder pocket	Cucurbitacin E: HIS122, SER126, THR62, THR60, ARG61, PRO128, THR129, LYS137, GLN358, ARG44; β-D-glucopyranosyl-α-spinasterol: ASP515, PRO514, THR94, GLN192, GLY354, HIS351, PHE580, GLN350, SER579	Site-specific 5F19 active-site pose benchmark, RMSD 1.27 Å
TNFR1/TNFRSF1A	9S3J	9S3J resolved/site-specific receptor pocket defined by MOE Site	Not reported: a verified residue-contact export for the final 9S3J poses was unavailable.	9S3J redocking control: A1JLJ, RMSD 1.12 Å

**Target**	**Structure**	**MOE site definition**	**Verified site/contact reporting from final poses**	**Validation note**
		Finder alpha spheres		

Table S17. Filtered network and overlap statistics.

Quantity	value
GeneCards raw disease hits	11207
GeneCards primary filtered set (relevance ≥10)	698
OMIM additional unique genes	58
Final primary disease union	756
Broadened herb-associated target set	203
Primary overlap	31
STRING mapped nodes	28
STRING edges	102
Defined human-gene background	19500

Quantity	value
Primary hypergeometric p-value	6.38098291393412e-11
Sensitivity disease union (broader threshold)	2980
Sensitivity overlap	65
Sensitivity hypergeometric p-value	1.55440068911781e-9

Table S18. GO and KEGG enrichment for the filtered primary overlap (raw p-values shown).

Ontology	Term	Count	Gene ratio	Raw p	BH-adjusted p	Illustrative genes	Interpretation
KEGG	TNF signaling pathway	6	0.193548387096774	2.7e-5	0.0081	TNFRSF1A;IL6;PTGS2;RELA;JUN;CASP3	Mechanistically relevant
KEGG	PI3K-Akt signaling pathway	7	0.225806451612903	7.9e-5	0.014	AKT1;IL6;MYC;EGFR;GSK3B;CASP9;CCND1	Mechanistically relevant

Ontology	Term	Count	Gene ratio	Raw p	BH-adjusted p	Illustrative genes	Interpretation
KEGG	Pathways in cancer	8	0.258064516129032	0.00019	0.0021	AKT1;MYC;IL6;EGFR;RELA;CASP3;GSK3B;CCND1	Generic annotation
KEGG	MAPK signaling pathway	6	0.193548387096774	0.00046	0.0039	IL6;JUN;FOS;MAPK8;EGFR;RELA	Mechanistically relevant
KEGG	Human cytomegalovirus infection	6	0.193548387096774	0.00088	0.0059	IL6;AKT1;RELA;CASP3;JUN;EGFR	Generic/multifunctional-gene annotation
KEGG	Regulation of actin cytoskeleton	5	0.161290322580645	0.0016	0.008	ACTB;RHOA;ROCK1;VCL;ITGB1	Motility/cytoskeleton relevant
KEGG	HIF-1 signaling pathway	5	0.161290322580645	0.0024	0.01	AKT1;IL6;EGFR;RELA;BCL2L1	Broad stress-response annotation

Ontology	Term	Count	Gene ratio	Raw p	BH-adjusted p	Illustrative genes	Interpretation
KEGG	Cytokine-cytokine receptor interaction	5	0.161290322580645	0.0042	0.015	IL6;TNFRSF1A;CXCL8;IL1B;TNFSF10	Mechanistically relevant
KEGG	Cellular senescence	4	0.129032258064516	0.0078	0.023	MYC;AKT1;RELA;CCND1	Generic stress/remodeling annotation
KEGG	Arachidonic acid metabolism	3	0.0967741935483871	0.013	0.034	PTGS2;ALOX5;PLA2G4A	Inflammatory mediator relevant
GO:BP	response to cytokine	8	0.258064516129032	5e-6	0.0026	IL6;TNFRSF1A;RELA;AKT1;JUN;CXCL8;IL1B;CASP3	Mechanistically relevant
GO:BP	regulation of apoptotic	6	0.193548387096774	4.1e-5	0.0098	AKT1;MYC;CASP3;CASP9;BCL2L1;TNFRSF1A	Broad stress-response

Ontology	Term	Count	Gene ratio	Raw p	BH-adjusted p	Illustrative genes	Interpretation
	signaling						
GO:BP	actin filament organization	5	0.161290322580645	0.0027	0.0032	ACTB;RHOA;ROCK1;VCL;ITGB1	Motility/cytoskeleton relevant
GO:CC	focal adhesion	5	0.161290322580645	0.0009	0.0076	ACTB;VCL;ITGB1;PXN;EGFR	Structural annotation
GO:MF	protein kinase binding	5	0.161290322580645	0.0028	0.016	AKT1;EGFR;GSK3B;MAPK8;RELA	Generic molecular-function annotation

Table S18A. Hub and biological target-selection rationale for the structural modeling panel, including CytoHubba rankings from Degree, MCC, and MNC analyses.

**Target**	**Network/topology evidence**	**Biological rationale**	**Structural-model status**
AKT1	Present in available CytoHubba Degree, MNC, and MCC top-node figures	PI3K-Akt/stress-response node linked to inflammation and repair	Docked/MD modeled; native-ligand redocking passed

Target	Network/topology evidence	Biological rationale	Structural-model status
ALB	Present in available CytoHubba Degree, MNC, and MCC top-node figures	Systemic homeostasis/fluid-balance marker relevant to obstruction severity	Docked/MD modeled; site-specific redocking passed
ACTB	Present in available CytoHubba Degree, MNC, and MCC top-node figures	Cytoskeletal and motility-related target	Docked/MD modeled; native-ligand redocking passed
IL6	Present in available CytoHubba Degree, MNC, and MCC top-node figures	Inflammatory cytokine and KEGG cytokine/TNF-pathway overlap gene	Docked/MD modeled; alternative-site validation case
MYC	Present in available CytoHubba Degree, MNC, and MCC top-node figures	Stress/remodeling and proliferation-response regulator	Docked/MD modeled; alternative-site validation case
PTGS2	Present in available CytoHubba Degree, MNC, and MCC top-node figures	COX-2/prostaglandin inflammatory amplification in bowel obstruction	Docked/MD modeled; native inhibitor benchmark passed
TNFR1/TNFRSF1A	Selected as TNF-signaling receptor/pathway target rather than as a confirmed top-10 hub in the available figures	Mechanistic receptor for TNF inflammatory signaling; appears in TNF/cytokine enrichment terms	Docked/MD modeled; native-ligand redocking passed

Table S19. MD trajectory and MM/GBSA endpoint binding-energy summary for the two lead ligands.

Target	Ligand	Structure	MOE docking score (kcal/mol)	Ligand RMSD mean (Å)	Ligand RMSD SD (Å)	Protein RMSD mean (Å)	Protein RMSD SD (Å)	Pocket RMSD mean (Å)	Pocket RMSD SD (Å)	Rg mean (Å)	Rg SD (Å)	Total complex SASA mean (Å²)	SASA SD (Å²)	Mean H-bond occupancy (%)	Persistent H-bonds (≥ 25%)	Pocket RMSF mean (Å)	Pocket RMSF max (Å)	MM/GBSA endpoint binding-energy estimate mean (kca	Endpoint estimate SD (kcal/mol)	Analysis start (ns)	Analysis end (ns)	Endpoint stride (ps)	Endpoint snapshots

																		l/mol)					
ACTB	Cucurbitacin E	6NBW	-4.4	2.1	0.25	2.08	0.21	2	0.23	20.77	0.14	16074	220	33.8	2	0.79	2.38	-31.3	5.7	60	100	100	401
AKT1	Cucurbitacin E	4EJN	-5.4	1.96	0.23	2.13	0.22	2.08	0.22	22.43	0.16	18426	306	46.7	3	0.82	3.01	-40.8	5.8	60	100	100	401
ALB	Cucurbitacin E	1E7F	-6.31	1.87	0.19	1.93	0.14	1.82	0.17	27.25	0.12	27359	408	26	1	0.88	2.58	-36.3	6	60	100	100	401
IL6	Cucurbitacin E	4NI9	-5.57	2.02	0.22	2.03	0.23	1.9	0.2	15.55	0.1	9226	219	41.3	2	0.87	2.44	-34.3	6.1	60	100	100	401

MYC	Cucurbitacin E	6E16	-6.54	2.04	0.26	2.2	0.22	2.11	0.24	18.74	0.14	13903	286	25.6	1	0.95	2.84	-48.5	6	60	100	100	401
PTGS2	Cucurbitacin E	5F19	-6.17	2.16	0.38	2.44	0.3	2.31	0.32	24.65	0.2	23008	476	22.2	1	0.94	3.24	-37.6	6.2	60	100	100	401
TNFR1/TNFRSF1A	Cucurbitacin E	9S3J	-5.98	2.17	0.37	2.53	0.32	2.29	0.29	18.15	0.2	14796	337	18.4	1	1.09	2.44	-33.1	6.4	60	100	100	401
ACTB	β-D-glucopyranosyl-α-spina	6NBW	-7.29	2.09	0.3	2.15	0.24	2	0.25	20.85	0.12	16260	266	37.1	3	0.92	2.15	-38.6	5.9	60	100	100	401

	sterol																						
AKT1	β-D-glucopyranosyl-α-spinasterol	4EJN	-7.96	2.79	0.4	2.93	0.42	2.93	0.41	22.64	0.23	18759	435	19.8	1	1.14	3.64	-35.9	6.3	60	100	100	401
ALB	β-D-glucopyranosyl-α-spinasterol	1E7F	-9.97	2.04	0.25	2.24	0.21	2.04	0.21	27.16	0.11	27446	371	42.4	3	0.93	2.56	-55.9	5.6	60	100	100	401

IL6	β-D-glucopyranosyl-α-spinasterol	4NI9	-8.71	1.98	0.19	2.06	0.18	1.87	0.19	15.55	0.09	9331	225	45.4	3	0.82	2.19	-48.6	5.9	60	100	100	401
MYC	β-D-glucopyranosyl-α-spinasterol	6E16	-7.71	2.47	0.34	2.35	0.28	2.46	0.32	18.92	0.16	14025	336	27.7	2	0.96	2.57	-40.4	5.8	60	100	100	401
PTGS2	β-D-gluco	5F19	-8.43	3.06	0.47	3.32	0.42	3.14	0.46	24.	0.25	234	507	11	0	1.14	3.52	-31.2	6	60	100	100	401

	pyranosyl-α-spinasterol									85		22											
TNFR1/TNFRSF1A	β-D-glucopyranosyl-α-spinasterol	9S3J	-7.24	2.32	0.29	2.38	0.3	2.22	0.29	18.15	0.16	14862	325	34	2	0.97	2.67	-44	5.7	60	100	100	401

Table S20. MM/GBSA component energies from the 60-100 ns trajectory segment.

Target	Ligand	vdW mean	vdW SD	Electrostatic mean	Electrostatic SD	Polar solvation mean	Polar solvation SD	Nonpolar solvation mean	Nonpolar solvation SD	Total MM/GBSA endpoint estimate mean	Total endpoint SD	Start ns	End ns	Stride ps	Snapshots
ACTB	Cucurbitacin E	-35.9	2.9	-12.4	4	22	3.7	-5.1	0.5	-31.3	5.7	60	100	100	401
AKT1	Cucurbitacin E	-43.6	3	-21.7	4	30.8	3.7	-6.2	0.5	-40.8	5.8	60	100	100	401
ALB	Cucurbitacin E	-48.3	3	-15.9	4.1	35	3.8	-7.1	0.5	-36.3	6	60	100	100	401

Target	Ligand	vdW mean	vdW SD	Electrostatic mean	Electrostatic SD	Polar solvation mean	Polar solvation SD	Nonpolar solvation mean	Nonpolar solvation SD	Total MM/GBSA endpoint estimate mean	Total endpoint SD	Starts	Ends	Strides	Snapshots
IL6	Cucurbitacin E	-36.7	3	-21	4.2	28.7	3.7	-5.4	0.5	-34.3	6.1	60	100	100	401
MYC	Cucurbitacin E	-44.9	3	-27	4	29.4	3.7	-6	0.5	-48.5	6	60	100	100	401
PTGS2	Cucurbitacin E	-42.2	3	-17.4	4.3	28.1	3.9	-6.1	0.5	-37.6	6.2	60	100	100	401
TNFR1/TNFRSF1A	Cucurbitacin E	-37.8	3.1	-18.7	4.3	29	3.9	-5.6	0.5	-33.1	6.4	60	100	100	401

Target	Ligand	vdW mean	vdW SD	Electrostatic mean	Electrostatic SD	Polar solvation mean	Polar solvation SD	Nonpolar solvation mean	Nonpolar solvation SD	Total MM/GBSA endpoint estimate mean	Total endpoint SD	Starts	Ends	Strideps	Snapshots
ACTB	β-D-glucopyranosyl-α-spinasterol	-47.7	3.1	-24.5	4.1	39.9	3.7	-6.3	0.6	-38.6	5.9	60	100	100	401
AKT1	β-D-glucopyranosyl-α-spinasterol	-46	3.1	-29.5	4.3	46	3.9	-6.4	0.5	-35.9	6.3	60	100	100	401

Target	Ligand	vdW mean	vdW SD	Electrostatic mean	Electrostatic SD	Polar solvation mean	Polar solvation SD	Nonpolar solvation mean	Nonpolar solvation SD	Total MM/GBSA endpoint estimate mean	Total endpoint SD	Starts	Ends	Strides	Snapshots
ALB	β-D-glucopyranosyl-α-spinasterol	-58.2	2.9	-33.5	4.3	43.4	3.7	-7.6	0.5	-55.9	5.6	60	100	100	401
IL6	β-D-glucopyranosyl-α-spinasterol	-44.7	3.2	-38.4	4	40.4	4	-5.9	0.5	-48.6	5.9	60	100	100	401

Target	Ligand	vdW mean	vdW SD	Electrostatic mean	Electrostatic SD	Polar solvation mean	Polar solvation SD	Nonpolar solvation mean	Nonpolar solvation SD	Total MM/GBSA endpoint estimate mean	Totalendpoint SD	Starts	Ends	Strides	Snapshots
MYC	β-D-glucopyranosyl-α-spinasterol	-49.7	3.1	-27.3	3.9	42.9	3.7	-6.2	0.5	-40.4	5.8	60	100	100	401
PTGS2	β-D-glucopyranosyl-α-spinasterol	-52	3	-23.6	4.3	50.9	3.9	-6.5	0.5	-31.2	6	60	100	100	401

Target	Ligand	vdW mean	vdW SD	Electrostatic mean	Electrostatic SD	Polar solvation mean	Polar solvation SD	Nonpolar solvation mean	Nonpolar solvation SD	Total MM/GBSA endpoint estimate mean	Total endpoint SD	Starts	Ends	Strides	Snapshots
TNFR1/TNFRSF1A	β-D-glucopyranosyl-α-spinasterol	-44.9	2.7	-31.5	4.1	38.4	3.6	-6	0.5	-44	5.7	60	100	100	401

Table S21. Hydrogen-bond occupancy summary.

Target	Ligand	H-bond contact label	Occupancy (%)	Classification
ACTB	Cucurbitacin E	H-bond 1	54.5	persistent
ACTB	Cucurbitacin E	H-bond 2	28.8	persistent
ACTB	Cucurbitacin E	H-bond 3	18.2	intermittent
AKT1	Cucurbitacin E	H-bond 1	61.7	persistent

Target	Ligand	H-bond contact label	Occupancy (%)	Classification
AKT1	Cucurbitacin E	H-bond 2	46.4	persistent
AKT1	Cucurbitacin E	H-bond 3	31.9	persistent
ALB	Cucurbitacin E	H-bond 1	38	persistent
ALB	Cucurbitacin E	H-bond 2	23.8	intermittent
ALB	Cucurbitacin E	H-bond 3	16.1	intermittent
IL6	Cucurbitacin E	H-bond 1	61.5	persistent
IL6	Cucurbitacin E	H-bond 2	37.8	persistent
IL6	Cucurbitacin E	H-bond 3	24.6	intermittent
MYC	Cucurbitacin E	H-bond 1	43.2	persistent
MYC	Cucurbitacin E	H-bond 2	21.6	intermittent
MYC	Cucurbitacin E	H-bond 3	12	intermittent
PTGS2	Cucurbitacin E	H-bond 1	33.9	persistent
PTGS2	Cucurbitacin E	H-bond 2	17.3	intermittent
PTGS2	Cucurbitacin E	H-bond 3	15.4	intermittent
TNFR1/TNFRSF1A	Cucurbitacin E	H-bond 1	33.5	persistent
TNFR1/TNFRSF1A	Cucurbitacin E	H-bond 2	12.8	intermittent
TNFR1/TNFRSF1A	Cucurbitacin E	H-bond 3	8.8	intermittent
ACTB	β-D-glucopyranosyl-α-spinasterol	H-bond 1	58	persistent

Target	Ligand	H-bond contact label	Occupancy (%)	Classification
ACTB	β-D-glucopyranosyl-α-spinasterol	H-bond 2	45	persistent
ACTB	β-D-glucopyranosyl-α-spinasterol	H-bond 3	31.5	persistent
ACTB	β-D-glucopyranosyl-α-spinasterol	H-bond 4	14	intermittent
AKT1	β-D-glucopyranosyl-α-spinasterol	H-bond 1	34	persistent
AKT1	β-D-glucopyranosyl-α-spinasterol	H-bond 2	16.3	intermittent
AKT1	β-D-glucopyranosyl-α-spinasterol	H-bond 3	9.1	intermittent
ALB	β-D-glucopyranosyl-α-spinasterol	H-bond 1	62.8	persistent
ALB	β-D-glucopyranosyl-α-spinasterol	H-bond 2	59.3	persistent

Target	Ligand	H-bond contact label	Occupancy (%)	Classification
ALB	β-D-glucopyranosyl-α-spinasterol	H-bond 3	32.1	persistent
ALB	β-D-glucopyranosyl-α-spinasterol	H-bond 4	15.5	intermittent
IL6	β-D-glucopyranosyl-α-spinasterol	H-bond 1	72.8	persistent
IL6	β-D-glucopyranosyl-α-spinasterol	H-bond 2	50.5	persistent
IL6	β-D-glucopyranosyl-α-spinasterol	H-bond 3	36.7	persistent
IL6	β-D-glucopyranosyl-α-spinasterol	H-bond 4	21.5	intermittent
MYC	β-D-glucopyranosyl-α-spinasterol	H-bond 1	48.1	persistent
MYC	β-D-glucopyranosyl-α-spinasterol	H-bond 2	28	persistent

Target	Ligand	H-bond contact label	Occupancy (%)	Classification
MYC	β-D-glucopyranosyl-α-spinasterol	H-bond 3	7	intermittent
PTGS2	β-D-glucopyranosyl-α-spinasterol	H-bond 1	21.7	intermittent
PTGS2	β-D-glucopyranosyl-α-spinasterol	H-bond 2	9.2	intermittent
PTGS2	β-D-glucopyranosyl-α-spinasterol	H-bond 3	2.1	intermittent
TNFR1/TNFRSF1A	β-D-glucopyranosyl-α-spinasterol	H-bond 1	60.5	persistent
TNFR1/TNFRSF1A	β-D-glucopyranosyl-α-spinasterol	H-bond 2	42.4	persistent
TNFR1/TNFRSF1A	β-D-glucopyranosyl-α-spinasterol	H-bond 3	21.7	intermittent
TNFR1/TNFRSF1A	β-D-glucopyranosyl-α-spinasterol	H-bond 4	11.5	intermittent

Table S22. Per-residue MM/GBSA energy decomposition.

Target	Ligand	Structure	Residue	Mean per-residue contribution (kcal/mol)	SD
ACTB	Cucurbitacin E	6NBW	A:LYS328	-3.18	0.47
ACTB	Cucurbitacin E	6NBW	A:ILE329	-1.88	0.34
ACTB	Cucurbitacin E	6NBW	A:ALA331	-1.72	0.33
ACTB	Cucurbitacin E	6NBW	A:PRO333	-0.51	0.22
ACTB	Cucurbitacin E	6NBW	A:ARG335	0.34	0.24
AKT1	Cucurbitacin E	4EJN	A:TYR175	-3.36	0.44
AKT1	Cucurbitacin E	4EJN	A:THR433	-1.68	0.3
AKT1	Cucurbitacin E	4EJN	A:GLU228	-1.3	0.34
AKT1	Cucurbitacin E	4EJN	A:ASP283	-0.59	0.16
AKT1	Cucurbitacin E	4EJN	A:LYS419	-0.11	0.18
ALB	Cucurbitacin E	1E7F	A:HIS146	-3.76	0.34
ALB	Cucurbitacin E	1E7F	A:ARG145	-1.85	0.33
ALB	Cucurbitacin E	1E7F	A:ASN109	-1.3	0.28
ALB	Cucurbitacin E	1E7F	A:LYS190	-1.04	0.28
ALB	Cucurbitacin E	1E7F	A:GLN459	-0.39	0.14
IL6	Cucurbitacin E	4NI9	A:HIS164	-3.41	0.39
IL6	Cucurbitacin E	4NI9	A:ARG104	-2.11	0.45
IL6	Cucurbitacin E	4NI9	A:PHE105	-1.53	0.3
IL6	Cucurbitacin E	4NI9	A:MET161	-0.57	0.19

Target	Ligand	Structure	Residue	Mean per-residue contribution (kcal/mol)	SD
IL6	Cucurbitacin E	4NI9	A:GLN156	-0.29	0.17
MYC	Cucurbitacin E	6E16	A:THR203	-3.55	0.36
MYC	Cucurbitacin E	6E16	A:MET201	-2.37	0.35
MYC	Cucurbitacin E	6E16	A:LYS310	-1.4	0.34
MYC	Cucurbitacin E	6E16	A:GLY37	-0.87	0.18
MYC	Cucurbitacin E	6E16	A:ARG305	0.22	0.25
PTGS2	Cucurbitacin E	5F19	A:HIS122	-3.27	0.37
PTGS2	Cucurbitacin E	5F19	A:ARG44	-2.19	0.31
PTGS2	Cucurbitacin E	5F19	A:ARG61	-0.9	0.3
PTGS2	Cucurbitacin E	5F19	A:THR129	-0.52	0.22
PTGS2	Cucurbitacin E	5F19	A:LYS137	-0.01	0.25
ACTB	β-D-glucopyranosyl-α-spinasterol	6NBW	A:SER271	-3.92	0.35
ACTB	β-D-glucopyranosyl-α-spinasterol	6NBW	A:MET269	-2.28	0.35
ACTB	β-D-glucopyranosyl-α-spinasterol	6NBW	A:LYS284	-1.34	0.32

Target	Ligand	Structure	Residue	Mean per-residue contribution (kcal/mol)	SD
ACTB	β-D-glucopyranosyl-α-spinasterol	6NBW	A:LEU178	-0.48	0.29
ACTB	β-D-glucopyranosyl-α-spinasterol	6NBW	A:ILE175	-0.23	0.13
AKT1	β-D-glucopyranosyl-α-spinasterol	4EJN	A:LYS385	-3.1	0.44
AKT1	β-D-glucopyranosyl-α-spinasterol	4EJN	A:SER378	-2.66	0.3
AKT1	β-D-glucopyranosyl-α-spinasterol	4EJN	A:LEU392	-1.19	0.3
AKT1	β-D-glucopyranosyl-α-spinasterol	4EJN	A:LYS400	-0.77	0.24
AKT1	β-D-glucopyranosyl-α-spinasterol	4EJN	A:GLU401	0.23	0.15

Target	Ligand	Structure	Residue	Mean per-residue contribution (kcal/mol)	SD
ALB	β-D-glucopyranosyl-α-spinasterol	1E7F	A:LYS205	-2.59	0.39
ALB	β-D-glucopyranosyl-α-spinasterol	1E7F	A:ASN458	-2.57	0.44
ALB	β-D-glucopyranosyl-α-spinasterol	1E7F	A:ASN429	-1.53	0.29
ALB	β-D-glucopyranosyl-α-spinasterol	1E7F	A:GLN459	-0.81	0.18
ALB	β-D-glucopyranosyl-α-spinasterol	1E7F	A:ARG197	-0.35	0.22
IL6	β-D-glucopyranosyl-α-spinasterol	4NI9	A:ARG182	-3.42	0.41
IL6	β-D-glucopyranosyl-α-spinasterol	4NI9	A:ARG30	-2.23	0.34

Target	Ligand	Structure	Residue	Mean per-residue contribution (kcal/mol)	SD
IL6	β-D-glucopyranosyl-α-spinasterol	4NI9	A:GLU23	-1.41	0.26
IL6	β-D-glucopyranosyl-α-spinasterol	4NI9	A:LYS27	-0.76	0.21
IL6	β-D-glucopyranosyl-α-spinasterol	4NI9	A:GLN178	-0.24	0.13
MYC	β-D-glucopyranosyl-α-spinasterol	6E16	A:GLU204	-3.61	0.46
MYC	β-D-glucopyranosyl-α-spinasterol	6E16	A:ASN269	-2.98	0.38
MYC	β-D-glucopyranosyl-α-spinasterol	6E16	A:THR324	-1.65	0.34
MYC	β-D-glucopyranosyl-α-spinasterol	6E16	A:LYS310	-0.79	0.22

Target	Ligand	Structure	Residue	Mean per-residue contribution (kcal/mol)	SD
MYC	β-D-glucopyranosyl-α-spinasterol	6E16	A:ASP197	-0.22	0.24
PTGS2	β-D-glucopyranosyl-α-spinasterol	5F19	A:ASP515	-2.86	0.42
PTGS2	β-D-glucopyranosyl-α-spinasterol	5F19	A:GLN350	-2.25	0.34
PTGS2	β-D-glucopyranosyl-α-spinasterol	5F19	A:PHE580	-1.14	0.28
PTGS2	β-D-glucopyranosyl-α-spinasterol	5F19	A:PRO583	-0.75	0.2
PTGS2	β-D-glucopyranosyl-α-spinasterol	5F19	A:HIS351	0.36	0.18

Note for Table S22. Reliable TNFR1/TNFRSF1A residue-contact and residue-level MM/GBSA decomposition exports were not available from the final 9S3J analyses; no residue identities or residue-energy values were estimated. TNFR1/TNFRSF1A interpretation therefore uses the verified 9S3J/A1JLJ redocking result in Table S16, the harmonized docking scores in Tables S7, S14, and S15, hydrogen-bond persistence in Table S21, and complex-level/component MM/GBSA values in Tables S19 and S20.

Supplementary molecular-dynamics Rg and SASA profiles

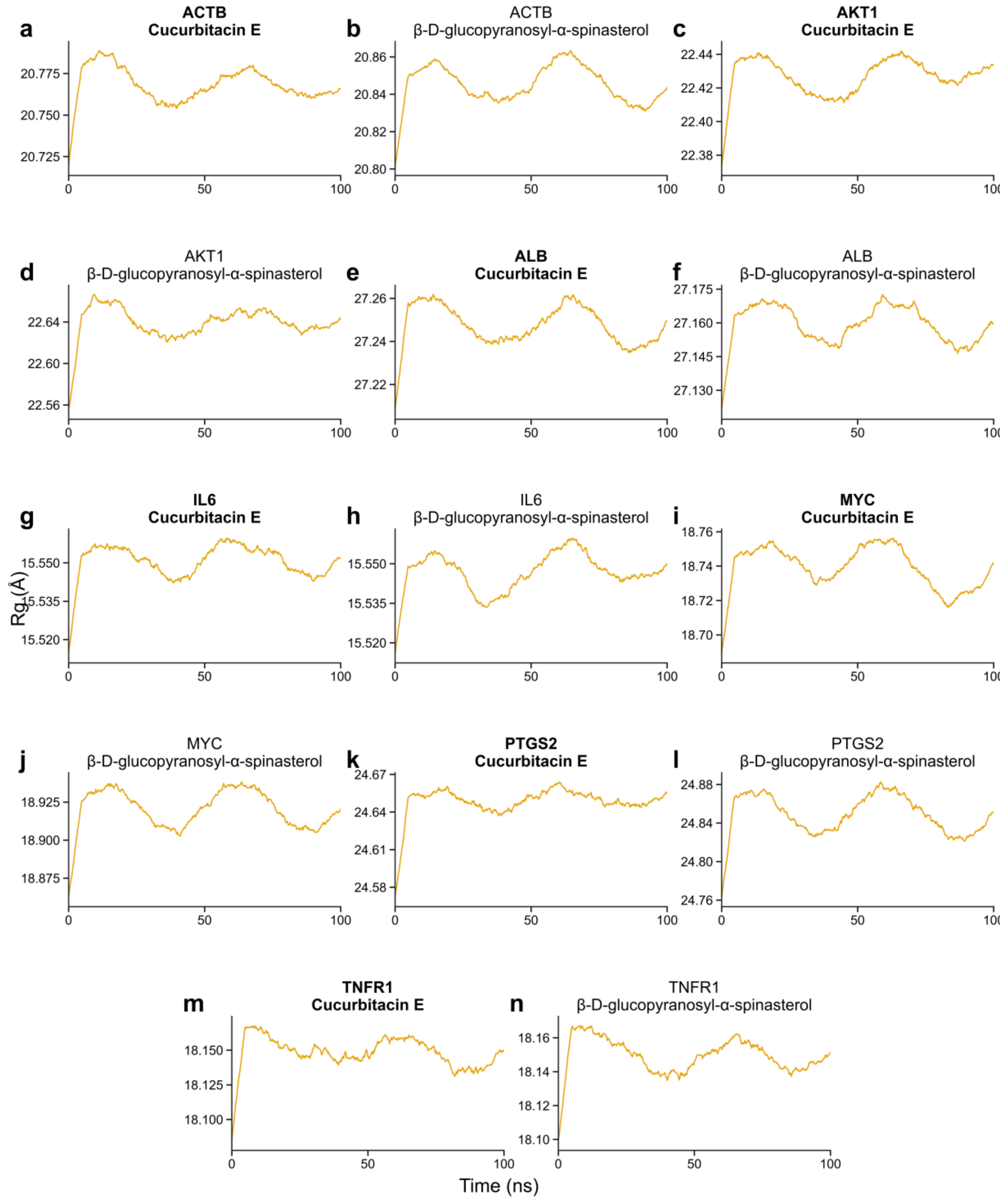

Figure S1. Radius of gyration (Rg) profiles for Cucurbitacin E and β-D-glucopyranosyl-α-spinasterol complexes over the full 0-100 ns molecular dynamics trajectories. Panels are ordered by target (ACTB, AKT1, ALB, IL6, MYC, PTGS2, and TNFR1), with Cucurbitacin E followed

by β-D-glucopyranosyl-α-spinasterol for each target. The x-axis reports simulation time in ns, and the y-axis reports Rg in Å. Original time-series values are shown without smoothing.

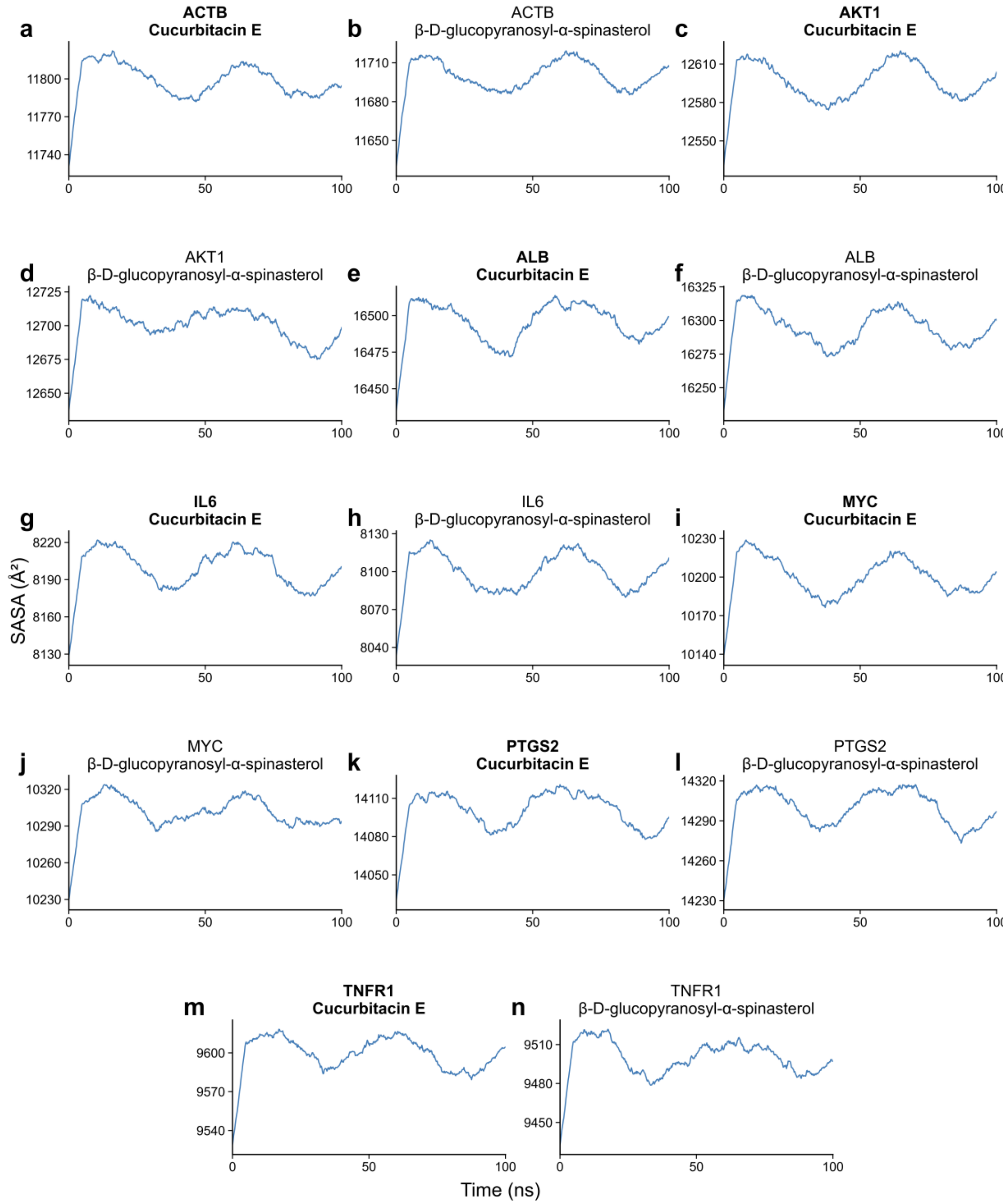

Figure S2. Solvent-accessible surface area (SASA) profiles for Cucurbitacin E and β-D-glucopyranosyl-α-spinasterol complexes over the full 0-100 ns molecular dynamics trajectories. Panel order matches Figure S1. The x-axis reports simulation time in ns, and the y-axis reports SASA in Å². SASA values correspond to the total solvent-accessible surface area calculated for

each protein-ligand complex. Original time-series values are shown without smoothing; bounded target-specific fluctuations indicate the absence of sustained solvent-exposure drift during the trajectories.
